# DszA Catalyzes C–S Bond Cleavage through N^5^–Hydroperoxyl Formation

**DOI:** 10.1021/acs.jcim.4c00301

**Published:** 2024-04-29

**Authors:** Pedro Ferreira, Rui P. P. Neves, Filipa P. Miranda, Ana V. Cunha, Remco W. A. Havenith, Maria J. Ramos, Pedro A. Fernandes

**Affiliations:** † LAQV,REQUIMTE, Departamento de Química e Bioquímica, Faculdade de Ciências, Universidade do Porto, Rua do Campo Alegre, s/n, Porto 4169-007, Portugal; ‡ Department of Chemistry, University of Antwerp, Groenenborgerlaan 171, Antwerp 2000, Belgium; § Stratingh Institute for Chemistry and Zernike Institute for Advanced Materials, University of Groningen, Nijenborgh 4, Groningen 9747 AG, The Netherlands; ∥ Ghent Quantum Chemistry Group, Department of Chemistry, Ghent University, Krijgslaan 281 (S3), Ghent B-9000, Belgium

## Abstract

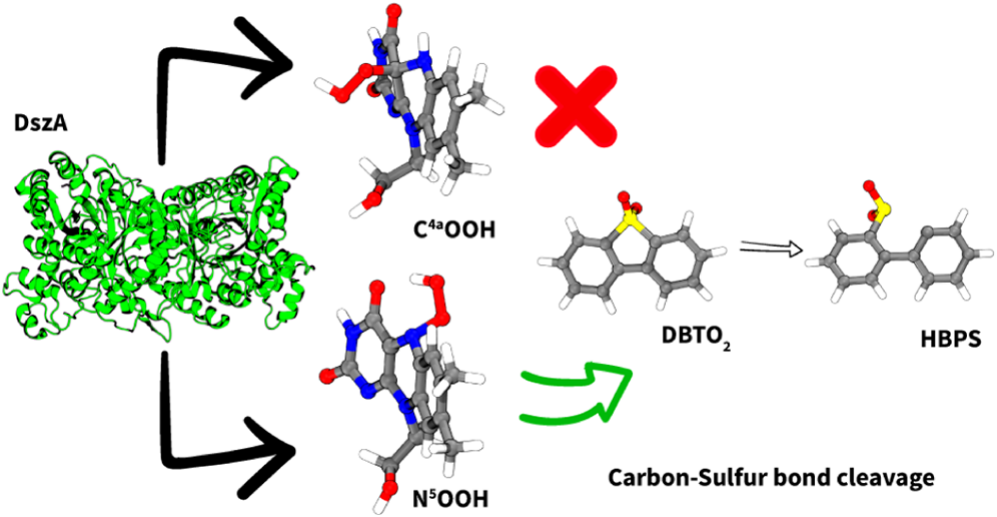

Due to its detrimental impact on human health and the environment, regulations demand ultralow sulfur levels on fossil fuels, in particular in diesel. However, current desulfurization techniques are expensive and cannot efficiently remove heteroaromatic sulfur compounds, which are abundant in crude oil and concentrate in the diesel fraction after distillation. Biodesulfurization *via* the four enzymes of the metabolic 4S pathway of the bacterium *Rhodococcus erythropolis* (DszA-D) is a possible solution. However, the 4S pathway needs to operate at least 500 times faster for industrial applicability, a goal currently pursued through enzyme engineering. In this work, we unveil the catalytic mechanism of the flavin monooxygenase DszA. Surprisingly, we found that this enzyme follows a recently proposed atypical mechanism that passes through the formation of an N^5^OOH intermediate at the *re* side of the cofactor, aided by a well-defined, predominantly hydrophobic O_2_ pocket. Besides clarifying the unusual chemical mechanism of the complex DszA enzyme, with obvious implications for understanding the puzzling chemistry of flavin-mediated catalysis, the result is crucial for the rational engineering of DszA, contributing to making biodesulfurization attractive for the oil refining industry.

## Introduction

Despite the long known adverse effects on the environment and human health caused by fossil fuel burning, our society is still overly dependent on fossil fuels for crucial activities. Ideally, fossil fuels should be entirely replaced by the increasing number of greener alternatives for energy production, but the demand for crude oil is still high and expected to rise in the near future.^1^ For this reason, as long as fossil fuels are used, we must mitigate their harmful effects.

**Figure 1:**
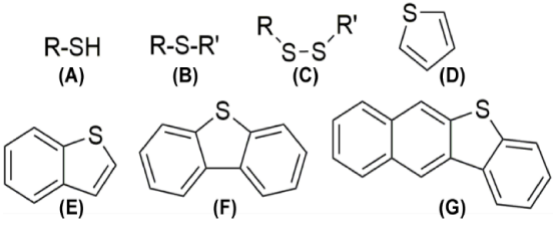
Scaffold of the main sulfur compounds present in the heavy crude oil. They include (A) thiols, (B) thioethers, (C) disulfides, (D) thiophenes, (E) benzothiophenes, (F) dibenzothiophenes, and (G) benzonaphthothiophenes. Reproduced from ref^2^ with permission from the Royal Society of Chemistry [Copyright 2020 Royal Society of Chemistry].

Besides carbon and hydrogen, sulfur is the most abundant element in crude oil, generally ranging between 0.1 and 6% (w/w) depending on its origin.^3^ Sulfur can be found in organic compounds such as thiols, sulfides, disulfides, thiolanes, thiophenes, benzothiophenes, dibenzothiophenes (DBT), and benzonaphthothiophenes (Figure 1). After crude oil distillation, DBT and its derivatives can account for up to 70% of the sulfur in the diesel fraction.^4,5^ Crude oil is classified as sweet or sour as the sulfur concentration is below or above 0.5%.^4^ One of the many harmful effects caused by the combustion of crude-oil products originates precisely from the presence of sulfur-containing molecules, which leads to the emission of hazardous gases such as H_2_S and SO_2_, which are ultimately responsible for acid rains.^6^ This motivated policymakers in several countries to demand ultralow concentrations of sulfur in fossil fuels (below 15 and 10 ppm in the USA and EU, respectively).^7,8^ However, the economic and environmental cost to desulfurize crude oil is very high since the desulfurization method commonly used (hydrodesulfurization, HDS) requires a very high temperature and pressure (>400 °C and 250 psi) and produces hazardous byproducts, such as H_2_S, and massive amounts of CO_2_; naturally, the higher the sulfur content is, the higher the hazards and costs will be.^9,10^ Unfortunately, most crude oil reservoirs under exploration nowadays are composed of sour oil, and those that contain sweet oil are being depleted since they have been preferred for decades.^11^

**Scheme 1:**
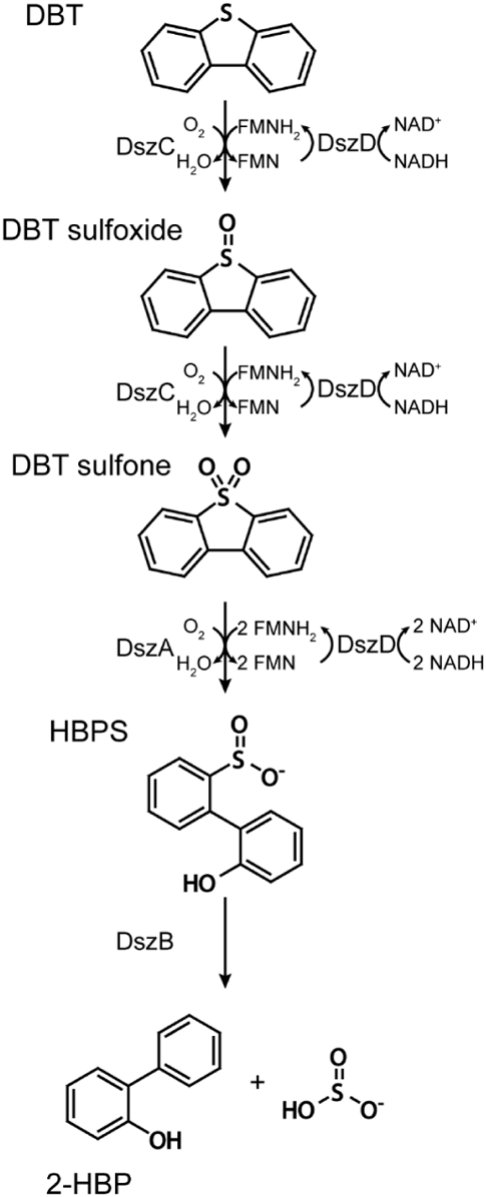
Schematic Representation of the Four Enzymatic Reactions of the 4S Pathway Reproduced from ref^11^ with permission from the Royal Society of Chemistry [Copyright 2020 Royal Society of Chemistry].

Biodesulfurization (BDS) has been proposed as a cheap and cleaner way to desulfurize oil, particularly for the removal of DBT and alkylated derivatives, which constitutes ca. 70% of sulfur content in diesel.^5,6,10,11,12^ This method is based on a metabolic pathway initially discovered in the *Rhodococcus erythropolis* bacterium, known as the 4S pathway, which employs four enzymes (DszA, DszB, DszC, and DszD) to remove sulfur from crude oil (Scheme 1). As the bacterium operates at room temperature and pressure, it avoids massive CO_2_ emissions, which is the primary appeal of the technique, in particular, if we remember that the oil refining industry is one of the principal CO_2_ producers in the world.

DszA and DszC are flavin mononucleotide (FMN) monooxygenases that oxidize the sulfur atom and ultimately break the C–S bond of DBT, with DszD providing the crucial FMNH_2_ cofactor for these enzymes. DszB is the final enzyme in the pathway responsible for the removal of SO_2_ from the carbon skeleton. The final products of the pathway are 2-hydroxybiphenyl (2-HBP) and HSO_3_-.^2^

Unfortunately, for industrial standards, the rate of desulfurization of BDS is too slow, which impedes its adoption by the oil refining industry.^13^ Therefore, there is a need to engineer the 4S enzymes to increase their catalytic rates, a subject gathering much attention.^14−16^ This requires extensive knowledge of their catalytic cycles, particularly a description of their catalytic mechanisms with an atomic level of detail. We have previously studied DszB,^17^ DszC,^18^ and DszD,^19,20^ and in this work, we focus on completing the overall mechanistic picture of the 4S pathway by deciphering the mechanism of DszA.

DszA (EC 1.14.14.22) is a flavin-dependent monooxygenase, the second to intervene in the 4S pathway after DszC. It is a member of group C flavin-dependent monooxygenases, which are characterized by depending on a separate flavin reductase to supply them with the reduced form of FMN. DszA oxidizes DBT-sulfone (DBTO_2_) into 2′-hydroxybiphenyl-2-sulfinate (HBPS), breaking one of the C−S bonds in DBTO_2_.^21^

There is a lack of structural and mechanistic information about this enzyme in the literature. However, an ortholog DBTO_2_ monooxygenase, BdsA from *Bacillus subtilis* WU-S2B, which shares 79% of sequence identity with DszA, was crystallized at a resolution of 2.8 Å in 2017 by Okai et al.^22^ This enzyme is reportedly active as a dimer, with each monomer exhibiting a TIM barrel fold and a sizable binding pocket to accommodate FMNH_2_, supplied by DszD, and the substrate.^22^

Bdsa and DszA possess similar functions, belong to the same group of the same superfamily, participate in the same catalytic step of the 4S pathway, possess FMN as the cofactor, and share a high sequence identity of 79%, and the conservation of residues at the active center is 91%. Therefore, the information gathered on the mechanistic study of BdsA is of great importance for understanding the DszA mechanism.

Su et al. managed to crystallize the BdsA complexed with oxidized FMN, and based on the obtained structure, it was suggested that BdsA only supplies the electrostatic environment for catalysis, but its residues do not intervene directly in the chemical reaction.^23^

**Scheme 2:**
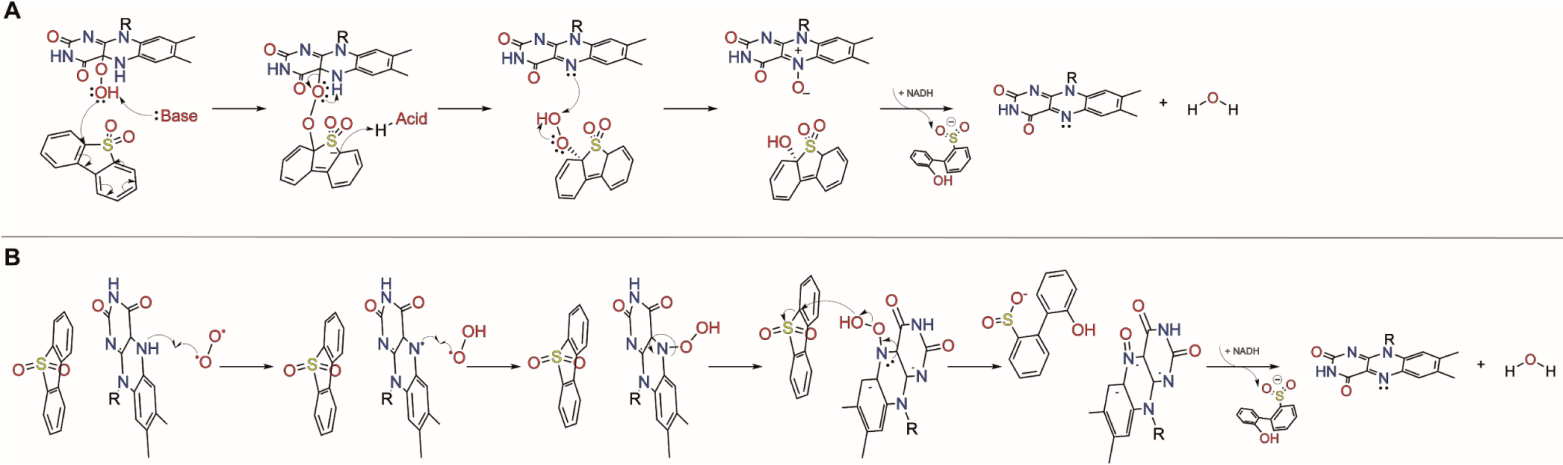
Reaction Mechanism for the Oxidation of DBT-Sulfone (DBTO_2_) to HBPS (A) The reaction mechanism for the oxidation of DBT-sulfone (DBTO_2_) to HBPS catalyzed by DszA as proposed by Adak and Begley. The reaction proceeds through a base-assisted formation of a peroxyhemiacetal intermediate between DBTO_2_ and the C^4a^-hydroperoxyflavin (C^4a^OOH), followed by an acid-assisted hydroperoxyl transfer to DBTO_2_ and subsequent formation of flavin N5-oxide to release HBPS. The flavin N5-oxide then requires an NADH cofactor for subsequent dehydration and formation of oxidized flavin. (B) Reaction mechanism for the oxidation of DBT-sulfone (DBTO_2_) to HBPS catalyzed by DszA through N^5^-hydroperoxyl formation as proposed for the flavoenzymes EncM and RutA. In this proposal, the O_2_ is activated and binds to the N^5^ at the *re*-side of the cofactor, followed by an inversion of the N^5^ atom that brings the OOH to the *si*-side, allowing the attack to the substrate.

Nonetheless, the catalytic mechanisms of BdsA and DszA are yet to be solidly determined. It was first studied by Adak and Begley in 2016 using liquid chromatography–mass spectrometry experiments.^24^ In their proposal, C–S cleavage of DBTO_2_ undergoes a base-assisted mechanism in which the distal oxygen of the C^4a^-hydroperoxyflavin intermediate, which is the common intermediate of the oxygen activation step of flavin-dependent monooxygenases, is deprotonated and becomes a nucleophile. The nucleophilic oxygen then attacks the carbon of the cleaving C–S bond to form a peroxyhemiacetal intermediate, ultimately leading to the transfer of the C^4a^-hydroperoxyl to the DBTO_2_ upon deprotonation of the N^5^ of the cofactor by its proximal oxygen. Finally, the transfer of the proximal oxygen to the N^5^ leads to the formation of a N^5^-oxide species, which requires NADH to restore the FMN cofactor to its oxidized state, and the HBPS product (Scheme 2A). Based on the X-ray structure of Su et al. for the BdsA homologue, the deprotonation of the reduced FMNH_2_ is facilitated by Ser139, which acts as a base. Then, a general molecular oxygen activation step should result in a flavin-C^4a^-hydroperoxide (C^4a^OOH), and the mechanism would then continue according to the mechanistic proposal by Adak and Begley.

Recent studies in other flavoenzymes, notably EncM, involved in 1,3-diketone oxidation, and RutA, which catalyzes the oxidative cleavage of the amide bond of uracil, unraveled an analogous route for the oxidative cleavage of inert carbon-hetero bonds. The route also includes the flavin-N^5^-oxide as an intermediate of the catalytic cycle.^25−28^ A pocket found at the *re*-side of the FMN in both EncM and RutA indicates that these enzymes accommodate O_2_ near N^5^,^26^ which promotes a hydrogen transfer from N^5^ to O_2_, yielding a superoxide and a semiquinone; afterward, a spin inversion of the superoxide from the triplet to the singlet state would allow its binding to the N^5^. In the next step, an inversion of the N^5^ atom would bring the OOH to the *si*-side of FMN (which is the location of the substrate pocket) into a near-attack conformation for the attack on DBTO_2_ (Scheme 2B).

The recent discovery of the flavin-N^5^-oxide intermediate opened new possibilities in the enzymology of flavin-dependent enzymes. In this work, we unveiled the catalytic mechanism of flavin monooxygenase DszA using hybrid quantum mechanics/molecular mechanics (QM/MM) calculations. Our results further contribute to the understanding of how flavin monooxygenases work and to the future development of efficient biocatalysts for crude oil desulfurization.

## Methods

### Modeling of the DszA:FMN Complex

The structure of DszA (UniProt ID: P54995) is currently unresolved. To study its catalytic mechanism, we used Swiss-Model^29^ to build a homology model of the enzyme using as a template the X-ray structure of BdsA (UniProt ID: Q8GRC7) from *Bacillus subtilis* in complex with FMN (PDB: 5XKD, resolution of 2.4 Å).^23^ This structure was chosen since it shares a sequence identity of 79% (91% at the active site) with DszA and catalyzes the same reaction. We built DszA as a tetramer with 13 772 atoms and used the homodimer composed by chains A and B to study the catalytic reaction, as this is the active form of the enzyme.^21^ The coordinates of FMN were transferred from the crystallographic structure of the complex BdsA:FMN.

The choice of the protonation states of the titratable residues was based on the p*K*_a_ estimation of propKa^30,31^ and visual inspection of hydrogen bond networks within titratable residues such as Asp, Glu, His or Lys. The latter was also used to determine the modeling of the His tautomers. All residues had standard protonation states at physiological pH. As for histidine residues, His116 and His316 were protonated at the δ- nitrogen while the remainder were protonated at the ε- nitrogen.

Based on the DszA:FMN complex, we modeled two different forms of flavin hydroperoxide, C^4a^OOH with the OOH at the *si* face and N^5^OOH with OOH at the *re* face of FMN (Figure S1). Force field parameters of C^4a^OOH were taken from Barbosa et al.,^18^ while N^5^OOH was parametrized using parameters from PARM10 and the second generation of the general AMBER force field^32^ (GAFF2) following the parametrization procedure of the AMBER force field. The atomic point charges were calculated at the HF/6-31G(d,p) level of theory with the restrained electrostatic potential (RESP) fitting.^33^ For the enzyme residues, we used the ff14SB. The complex was solvated in a rectangular 12 Å water box of TIP3P water molecules,^34^ and the system was neutralized with 46 Na^+^ ions (approximately 0.8 M).

The behavior of the modeled systems was inspected with conventional MD simulations (cMD), ran with Gromacs 2021.5.^35^ First, we conducted a three-step minimization procedure to optimize the modeled system: (1) energy minimization followed by 50 ps *NVT* simulation with harmonic restraints applied in the solute; (2) energy minimization with harmonic restraints applied in the backbone heavy atoms of DszA; and (3) energy minimization with no harmonic restraints applied. Afterward, we performed a three-step equilibration protocol to prepare the system for cMD simulations, encompassing (1) annealing of the system to 300 K at constant volume for 20 ps, followed by 30 ps at 300 K in the *NVT* ensemble; (2) a short simulation of 2 ns in the *NPT* ensemble (1 bar and 300 K) to relax the density of the solvent by applying harmonic restraints to the solute; and (3) an *NPT* simulation of 10 ns without restraints. Finally, a production of 100 ns with no applied restraints was conducted in the *NPT* ensemble (1 bar and 300 K). The velocity-rescaling thermostat^36^ was used in all MD simulations to keep the temperature at 300 K. To keep the pressure of the system at 1 bar in *NPT* simulations, the Berendsen barostat^37^ was used whenever harmonic restraints were applied; else, the Parrinello–Rahman barostat^38,39^ was used. A cutoff of 10 Å was used for the explicit calculation of nonbonded interactions, beyond which Coulomb interactions were treated with the particle-mesh Ewald summation method and Lennard-Jones interactions were truncated. The LINCS algorithm^40^ was used to constrain covalent bonds with hydrogen atoms in order to use an integration step of 2 fs. The details of the modeling are provided in the Supporting Information, Modeling of DszA:FMN section.

### Modeling of the DBTO_2_ Substrate into DszA

No structure of a catalytically competent DszA complex is available to date. The two available proposals pointing toward the C^4a^OOH-driven mechanism are based on molecular docking calculations by Su et al. on the ortholog BdsA:FMN complex^23^ and Neves et al. on a DszA:C^4a^OOH complex using the BdsA:FMN complex as a template.^2^ In light of the recent X-ray structure with PDB ID: 6TEG, put out by Matthews et al. for pyrimidine monooxygenase RutA (UniProt ID: P75898) in complex with O_2_ and its uracil substrate, an N^5^OOH-driven mechanism could also be possible.^25^ In any of these works, the substrate binds at the *si* face of FMN in an approximately parallel fashion and within 3–5 Å of the cofactor; the binding mostly involves contacts with aromatic or hydrophobic residues.^2,23,25^

To obtain the DszA:FMN:DBTO_2_ complex, the substrate, DBTO_2_, was built in Gaussview 5.0.8.^41^ Both the optimized coordinates and the atomic charges were obtained at the HF/6-31G(d,p) level of theory using the RESP approach. Force field parameters were obtained from GAFF2.

Molecular docking was performed using the minimized structures and the representative structure of the most populated cluster of the MD simulations of both DszA:C^4a^OOH and DszA:N^5^OOH complexes, using Autodock Vina^42^ or Gold^43^ and the corresponding genetic algorithms to generate the different poses of the substrate. A maximum of ten substrate poses were obtained for each complex, and the best ones underwent the same minimization and cMD protocols described above. The criteria used to select the best poses were the pose ranking and the short distance (3 to 5 Å) between the distal oxygen of flavin-hydroperoxide and the C_α1_ or C_α2_ atoms of DBTO_2_, as reported in literature.^18,25,44^ In both cases, the OOH was modeled at the *si* face of the FMN cofactor because that is the side where the cleavage of the C–S bond of DBTO_2_ should occur. More details can be found in the Supporting Information, Modeling DBT-sulfone binding.

Systems undergoing MD simulations were prepared as described in the Modeling of the DszA:FMN complex section in the Supporting Information. On a side note, we conjectured that, as for DszC, His316 could deprotonate the N^1^ of FMNH_2_*via* Ser139,^18,45^ and its protonation state upon DBTO_2_ binding could depend on whether a C^4a^OOH or N^5^OOH intermediate was formed. While during the formation of C^4a^OOH, the His316 could assist the protonation of O_2_ at the *si* face to form the hydroperoxyl radical binding C^4a^, as observed for the His391 of DszC,^18^ in the N^5^OOH pathway, the O_2_ would bind at the *re* face and deprotonate the N^5^, leaving His316 positively charged during the catalytic reaction and available to interact the sulfone moiety of DBTO_2_. Hence, His316 was protonated at the δ-position when modeling C^4a^OOH and in the positively charged form when modeling N^5^OOH. We then conducted a minimization procedure consisting of (1) energy minimization followed by 50 ps *NVT* simulation with harmonic restraints applied in the solute; (2) energy minimization with harmonic restraints applied in the backbone heavy atoms of DszA; (3) energy minimization with harmonic restraints applied in the solute residues other than those within 4.5 Å of the modeled FMN cofactor and DBTO_2_ substrate; and (4) energy minimization with no harmonic restraints applied. During the equilibration stage, in between the annealing/solvent density equilibration steps and the 10 ns unrestrained *NPT* cMD simulations, we performed an additional 10 ns cMD simulations in the *NPT* ensemble applying the same harmonic restraints in the solute residues other than those within 4.5 Å of the modeled FMN cofactor and DBTO_2_ substrate, as well as active site residues identified as relevant for either binding or catalysis (His20, Asp59, Ser139, His156, Arg159, Tyr160, and His316), to assess local active site rearrangements resulting from the modeling of C^4a^OOH/N^5^OOH and DBTO_2_. Final cMD productions then consisted of a single 100 ns replica in the *NPT* ensemble (300 K and 1 bar)—modeling details and analyzes are provided in the Modeling DBT-sulfone binding and the C^4a^OOH vs N^5^OOH model sections in the Supporting Information.

### Quantum Mechanics/Molecular Mechanics Calculations

To study the catalytic mechanism of DszA, we employed QM/MM calculations as we have done successfully before in many other enzymes.^17,18,46−49^ In particular, we used the QM/MM additive scheme as implemented in ORCA 5.0.3.^50,51^ Representative catalytically competent conformations were retrieved from the cMD simulations of the DszA:C^4a^OOH:DBTO_2_ and DszA:N^5^OOH:DBTO_2_ systems, after a cluster analysis based on the RMSD of the active site, using the GROMOS algorithm^52^ as implemented in Gromacs 2021.5. A 1.0 and a 1.4 Å cutoff were required for the restrained and unrestrained MD simulations, respectively. Representative structures from each of the dominant clusters were compared and the ones with, simultaneously, the most occupied DBTO_2_ binding pose and the lowest RMSD for both active site and DszA backbone relatively to the reference X-ray structure were chosen as templates to proceed for QM/MM calculations. A detailed analysis can be found in the C^4a^OOH vs N^5^OOH Model section in the Supporting Information.

**Figure 2:**
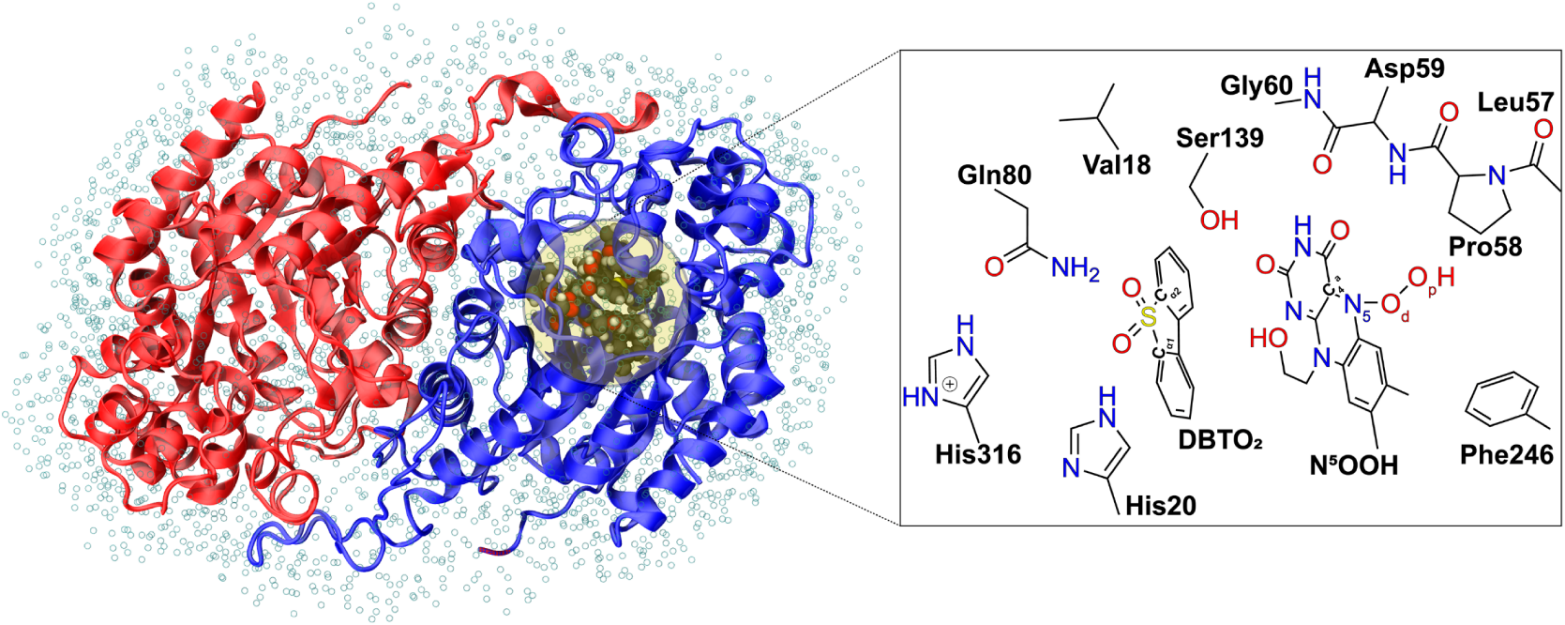
Representation of the QM/MM model used to study the catalytic mechanism of DszA. The QM layer was composed of 139 atoms and 19 link-atoms. It included the whole DBTO_2_, the isoalloxazine ring, and the hydroxyethyl of the ribitol tail bound to the N^10^ of the C^4a^- or the N^5^-hydroperoxyflavin intermediate (in the image it is the N^5^-hydroperoxyflavin that is represented), the side chains of His20, His316, Gln80, Val18, Ser139, Phe246 and the peptide composed by Pro58 and the backbones of Leu57, Asp59, and Gly60. In addition, three water molecules are present in the QM layer. The nomenclature used in the text for the atoms that are important for catalysis is shown

Two different QM/MM models were used to test the two main mechanistic hypotheses; one with the DszA:C^4a^OOH:DBTO_2_ complex and the other with the DszA:N^5^OOH:DBTO_2_ complex, each with 139 atoms assigned to the QM layer (with an overall neutral charge) and a total of 22 915 atoms. The active region includes all atoms of the QM layer and all atoms within 6 Å of the QM layer. We retained a 4 Å water cap surrounding the enzyme (Figure 2). The link-atom approach was used to complete the valence of the bonds cut by the boundary between the two layers. A total of 19 link-atoms were present in the system. The electrostatic interaction between layers was computed at QM level, using the electrostatic embedding scheme as implemented in ORCA 5.0.3.

The QM atoms were treated with the PBE0 DFT functional,^53^ as it performed well in a benchmark testing DFT functionals for similar reactions,^54^ Grimme’s D3BJ dispersion correction,^55^ and the def2-SVP basis set.^56^ Mechanistic steps were assessed through linear transit energy scans along tentative coordinates to obtain guess minima, which were then interpolated with the Climbing Image Nudged Elastic Band (CI-NEB) method implemented in ORCA 5.0.3, to obtain a guess structure for the transition state of each step from the maximum of the resulting minimum energy path. Guess transition states for each mechanistic step were then optimized to transition state with an eigenvector following an algorithm using a p-RFO step implemented in ORCA 5.0.3. The minima associated with the optimized transition states were obtained with Intrinsic Reaction Coordinate calculations, followed by geometry optimizations. The nature of the stationary states was confirmed by calculating the vibrational frequencies: all transition states displayed one imaginary frequency associated with the chemical step and minima had none. The Gibbs free energies were estimated by adding to the electronic energies the zero-point energy, the thermal energy at 298.15 K, and the entropy obtained by numeric rigid-rotor and harmonic-oscillator calculations. This method is based on the ideal gas statistical mechanics. This introduces an error in the entropy term since in solution the system is much more constrained. However, in this case, the Gibbs free energy profile is largely dominated by the enthalpy term, and thus, we consider the approximation carried out appropriate and not troublesome.

Final energies were obtained with single-point energy calculations using the highly accurate perturbatively corrected double-hybrid functional PWPB95,^57^ which is based on the Perdew–Wang (PW) GGA-exchange^58^ and the Becke95 (B95)^59^ meta-GGA-correlation functionals, and the def2-TZVPP basis set with the Coulomb-fitting auxiliary basis set def2/JK^56^ and the correlation fitting def2-TZVPP/C,^60^ to treat the QM layer. The Grimme’s D3BJ dispersion correction was also used. This double-hybrid functional excels in previously conducted benchmarks often exhibiting a higher accuracy than single-hybrids and the MP2 level.^57,61^

To check if the obtained energy profile is not dependent on the density functional, we repeated single-point energy calculations with the def2-TZVPP basis set with 7 different DFT functionals (PBE0, CAM-B3LYP, M06-2X, PW6B95, MPWB1K, PWPB95, and DSD-PBEB95). The values obtained are discussed Table S2 and Figure S22 .

Analyses of charge and density populations were conducted at the PBE0-D3BJ/def2-TZVPP:ff10 level, using a Mulliken population analysis, in order to discuss charge transfer and spin inversion processes.

## Results and Discussion

### Modeling of the Enzyme:Substrate Complex

The DszA:FMN complex was predicted using the sequence of DszA available in the UniProt entry P54995 and the X-ray structure of BdsA (PDB code: 5XKD) as a template. The best resulting model exhibited a QMEAN of −0.62 and a global quality estimation score (GMQE) of 0.91. Upon superimposing the model with the X-ray structure of BdsA, an RMSD of 0.13 Å over the backbone atoms was obtained, and a nearly identical active site conformation, preserving the interactions between the enzyme and FMN, was observed. MD simulations of the resulting complex also showed that the binding mode relative to that observed in the BdsA:FMN complex and the network of interactions between FMN and residues in the active site are conserved. The clustering analysis by the RMSD of the active site atoms of DszA and the comparison with the BdsA:FMN complex indicates that the isoalloxazine moiety of the FMN cofactor is stabilized in the active site through hydrogen bonds between the N^3^ and the O^4^ of the ring III and the backbone of Asp59 and between the O^2^ of the ring III and the Ser139-hydroxyl, as well as through hydrophobic contacts with the conserved Ala228, Leu230, and Phe246, whereas the ribitol tail established conserved hydrogen bonds between the phosphate tail and the backbones of Leu230-Ser231 or the side chain of His156, Tyr160, and Ser231 (Figure S5).

### DBT-Sulfone Modeling

The subsequent molecular docking calculations conducted to construct the catalytically active DszA:FMN:DBTO_2_ complex confirmed that the substrate docks at the *si* face of the isoalloxazine ring of FMN. The best substrate poses obtained for DszA:C^4a^OOH and DszA:N^5^OOH were found to be different (Figure S10). In the DszA:C^4a^OOH model, the OOH group partially occupies the binding pocket of the substrate, at the *si* face of the cofactor, which impedes the parallel alignment of the substrate with the cofactor. The distal oxygen of C^4a^OOH is 3.6 Å from C_α1_ and 4.6 Å from C_α2_ of DBTO2; one of the oxygens of the sulfone group of the substrate is accepting a hydrogen bond from the N^5^ hydrogen atom of the cofactor, and the other is interacting with His20. In the DszA:N^5^OOH model, the substrate and cofactor are closer, with their planes almost parallel. The distal oxygen of OOH stays farther from the C_α1_/C_α2_ atom of DBTO_2_ comparatively to the complex obtained in the DszA:C^4a^OOH model, since it is at the *re* face of the cofactor. The oxygen atoms of the sulfone group of the substrate make hydrogen bonds with His20 and Gln80.

**Figure 3:**
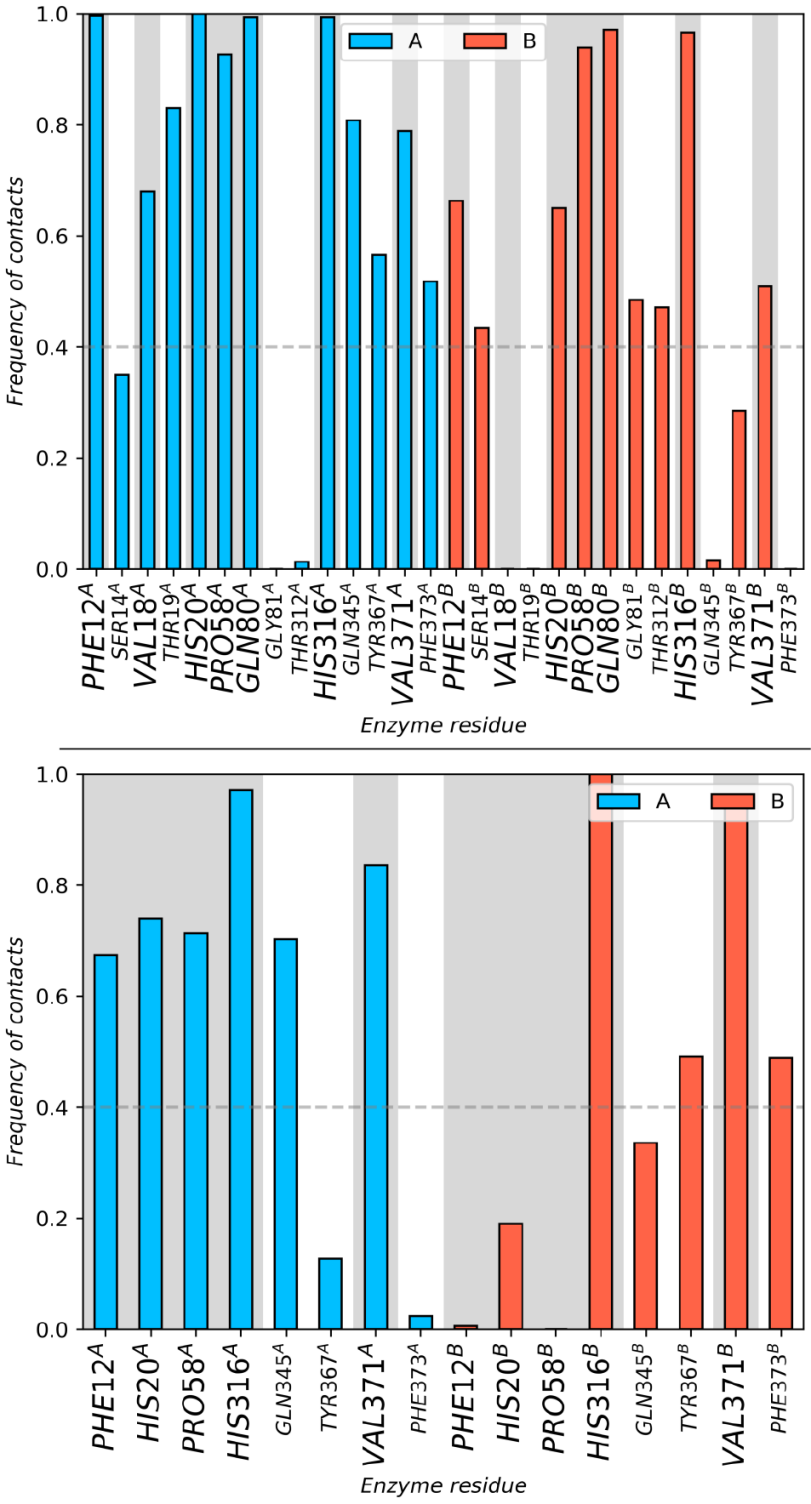
Contacts between heavy atoms of DBTO_2_ and DszA residues within 4 Å throughout the 100 ns unrestrained molecular dynamics simulations, for the DszA:C^4a^OOH complex (up) and the DszA:N^5^OOH complex (down). Residues labeled in larger font and highlighted with gray bars correspond to native contacts suggested from the starting docking pose.

In an attempt to explore binding modes for DBTO_2_ at the active site of DszA, the simulation of the enzyme:substrate complex encompassed two stages: a 10 ns simulation where only DszA residues beyond the active site were restrained in order to accommodate the C^4a^OOH/N^5^OOH cofactor and the DBTO_2_ substrate and a 100 ns simulation where the whole system was unrestrained. In such a way, we wanted to explore DBTO_2_ dynamics under different active site simulation conditions. Both simulations confirmed that the overall conformation of DszA and its active site were maintained (Figures S11,12), although the dominant DBTO_2_ pose slightly differed in each monomer. Nevertheless, we observed that most contacts from the docking-generated poses were preserved (Figure 3), regardless of the simulation conditions and the modeling of the FMN cofactor (either as C^4a^OOH or as N^5^OOH), which indicates that the DBTO_2_ binding mode should still resemble that of the docking.

A clustering analysis over the selection considered as active site (residues within 4.5 Å of the modeled FMN cofactor, the DBTO_2_ substrate, and residues His20, Asp59, Ser139, His156, Arg159, Tyr160, and His316, inclusively) provided one single cluster when DszA residues beyond the active side were restrained (Figure S13). The resulting DBTO_2_ pose and active site arrangement closely resembled the one from the docking calculations for both N^5^OOH (0.73 and 0.62 Å for each monomer, over all active site heavy atoms) and C^4a^OOH (0.44 and 0.62 Å for each monomer, over all active site heavy atoms). When the same analysis was extended to the unrestrained simulations (results summarized in Table S1 and Figure S15), the largest registered differences concerned the DBTO_2_ substrate and, more occasionally, the modeled FMN cofactor, as the RMSD of DszA residues varied between 0.63 and 1.39 Å relative to the reference minimized structure.

In particular, the largest differences to the minimized reference structures were registered for DBTO_2_ in the presence of the C^4a^OOH cofactor (heavy atom RMSD above 3 Å for more than 80% of the simulation), whereas, in the presence of N^5^OOH, the DBTO_2_ pose is preserved throughout over more than 90% of the simulation regardless of the DszA monomer. Nevertheless, the general binding mode of DBTO_2_ resembles that of the docking calculations.

**Figure 4:**
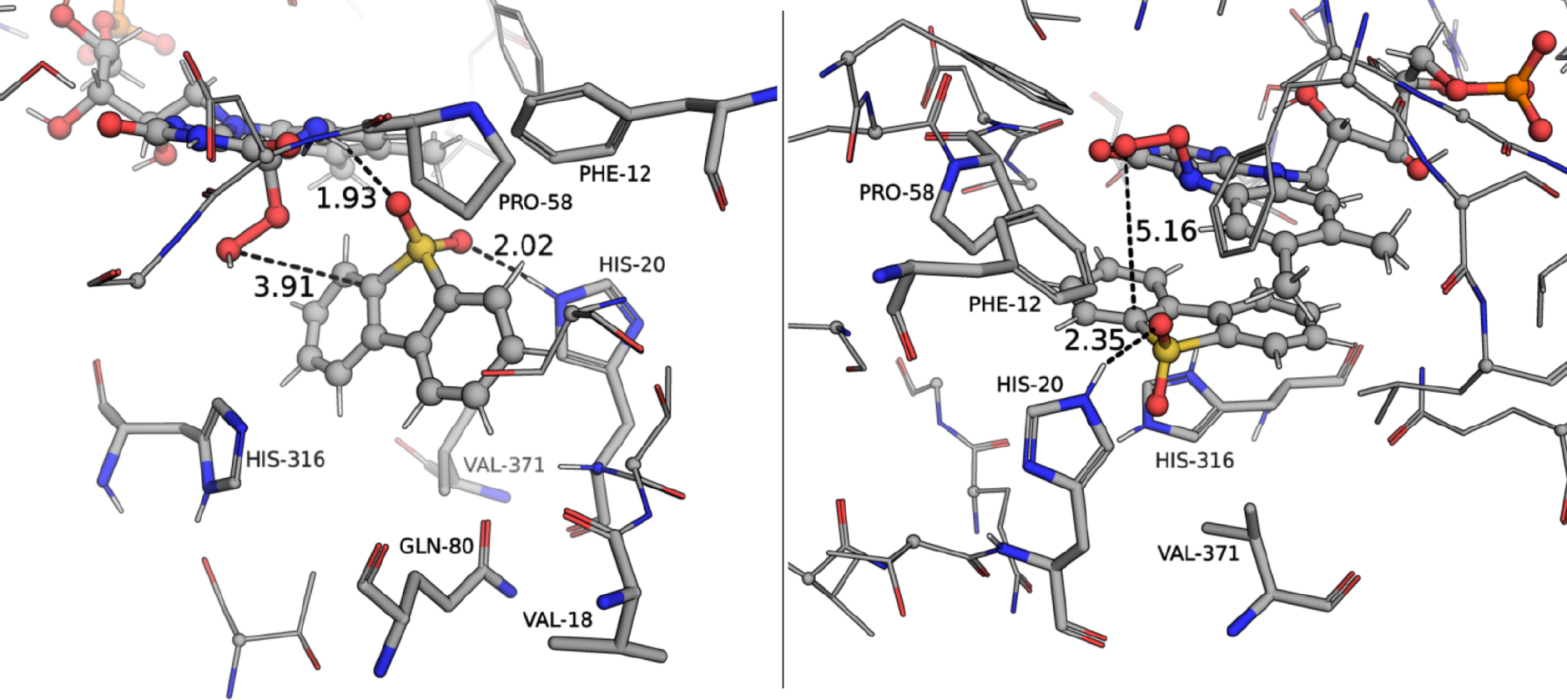
Active site of the representative structure of the cluster with the highest occupation for the DszA:C^4a^OOH complex (left) and the DszA:N^5^OOH complex (right). Residues identified as contacts in the docking calculations are shown in stick representation, and those conserved throughout the molecular dynamics simulations are labeled. The distance between the distal oxygen of C^4a^OOH/N^5^OOH and the nearest Cα of DBTO_2_ is also highlighted, as well as the conserved hydrogen bond between the sulfone of DBTO_2_ and the His20.

In both cases, the DBTO_2_ binding site is composed by Phe12, His20, Pro58, His316, Gln345, and Val371, but it exhibits quite different modes of interaction, as represented in Figure 4: in the presence of C^4a^OOH, DBTO_2_ is mostly kept in the active site through hydrogen bonds between its sulfone and His20 and the N^5^ of C^4a^OOH, whereas in the presence of N_5_OOH, DBTO_2_ is sandwiched between the isoalloxazine moiety of N^5^OOH and the His316 and conserves the hydrogen bond between its sulfone group and His20.

### Catalytic Mechanism

We obtained two plausible long-lasting DBTO_2_ poses, either considering C^4a^OOH or N^5^OOH, conserving features required for catalysis by DszA under the current proposed mechanistic hypotheses (there is at least one Cα of DBTO_2_ within 4–6 Å of the OOH group in the cofactor, Figure S16).^24,25^ As such, we considered two models, one for the DszA:C^4a^OOH:DBTO_2_ complex and another for the DszA:N^5^OOH:DBTO_2_ complex, to proceed for QM/MM calculations, and focused our calculations at the active site of chain A (where we registered the most stable DBTO_2_ poses, refer to Table S1). Following clustering analyzes based on the RMSD of the DszA active site, we chose the representative structure of the cluster with the most conserved DBTO_2_ binding pose considering restrained and unrestrained MD simulations, the highest cluster occupancy, and the lowest RMSD for the DszA backbone and active site. As such, the DszA:C^4a^OOH:DBTO_2_ model was built from the most occupied cluster of the whole unrestrained MD (Figure15, C^4a^OOH) and the DszA:N^5^OOH:DBTO_2_ model was built from the most occupied cluster of the active site unrestrained MD (Figure14, N^5^OOH).

### The C^4a^OOH Model

The energy profile obtained for the mechanistic hypothesis passing through a C^4a^OOH intermediate returned a very high energy barrier (above 30 kcal·mol^–1^), which is incompatible with the bacterium’s metabolism and does not match the experimentally measured reaction rate of 18.6 kcal·mol^–1 62^. Three tentative reaction coordinates were tested: the distance between the distal oxygen of C^4a^OOH and the C_α1_ atom of DBTO_2_; the distance between the proximal oxygen of C^4a^OOH and the C_α1_ atom of DBTO_2_; and the distance between the proton of OOH and His316. None led to the formation of a stable peroxyhemiacetal intermediate, as proposed in the literature. Instead, a simple OH transfer occurs from C^4a^OOH to the C_α1_ atom of DBTO_2_ (Figure S20). The only residue that could act as a catalytic base in the vicinity of C^4a^OOH and deprotonate OOH is His316. However, if the catalytic step is initiated with a proton transfer from OOH to His316, as His316 gets closer to OOH, it induces repulsive interactions with the cofactor:substrate complex, which prevents the formation of an energy minimum structure with a protonated His316 and C^4a^OO^–^. The observation is in line with those from other flavin monoxygenases, in which dioxygen quickly reduces to superoxide in the active site of the enzyme and spontaneously deprotonate a neighboring His, preventing C4_a_OO^–^ formation.^18,63,64^ Summarized results are included in the Mechanistic Attempts through the C^4a^OOH Model section in the Supporting Information.

### The N^5^OOH Model

**Figure 5:**
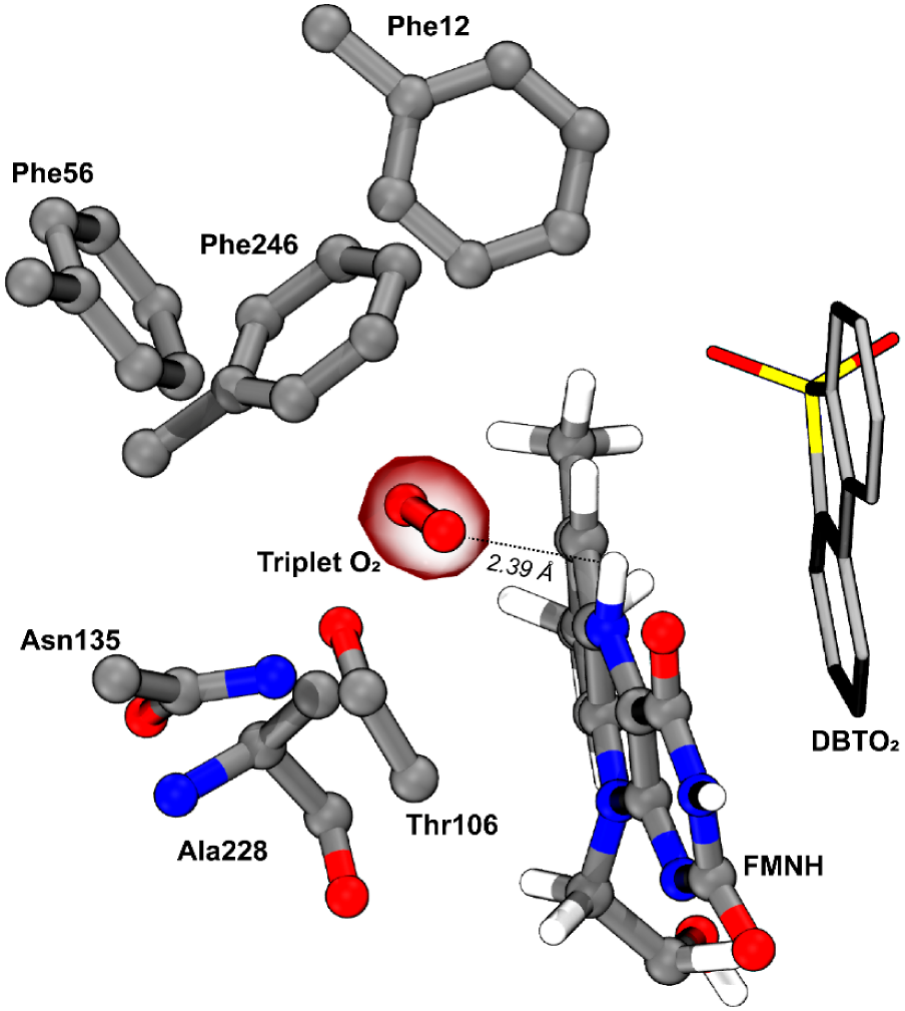
O_2_ binding pocket of DszA located on the *re* side of FMNH. Hydrogen atoms of the residues are not shown for clarity.

**Figure 6:**
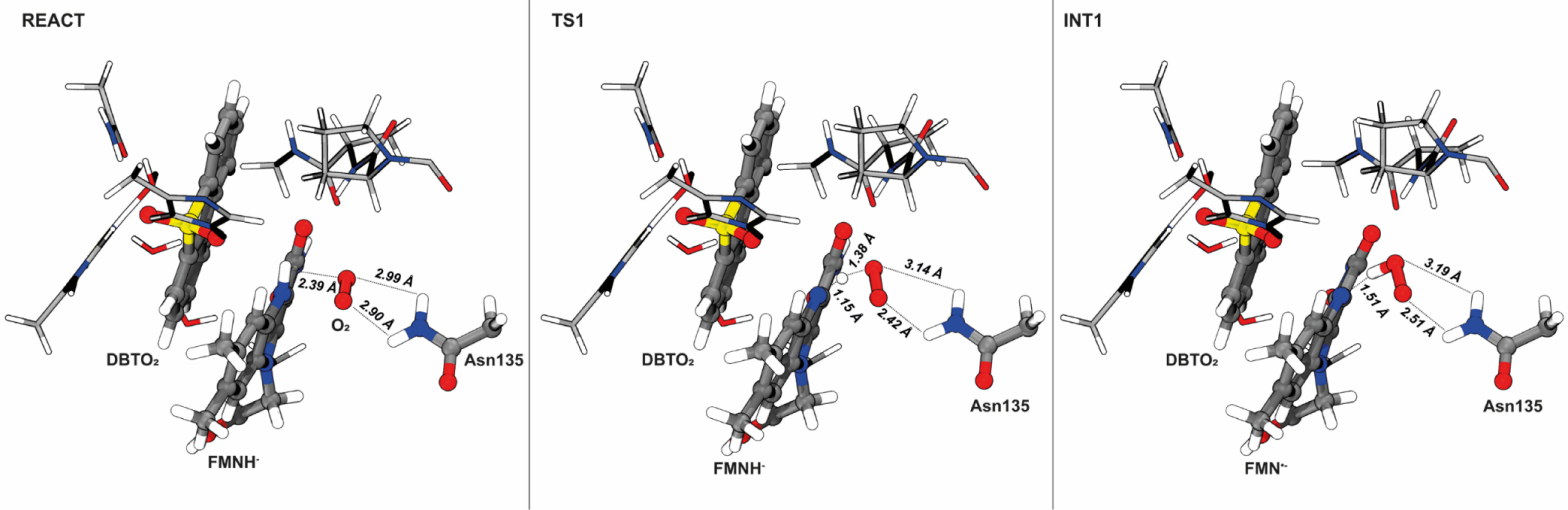
Representation of the stationary points of the first step of the catalytic mechanism of DszA. This step corresponds to the proton-coupled electron transfer from FMNH^−^ to the triplet dioxygen molecule, resulting in a semiquinone and a hydroperoxyl. The reaction coordinate (defined by the distance between the O_d_ and the H atom bonded to N^5^ and the lengths of the H-bonds between the hydroperoxyl and Asn135) are highlighted.

Using the N^5^OOH model, we found that the triplet dioxygen molecule stays stable in a neutral and mostly hydrophobic pocket near the N^5^ atom of FMNH at the *re* side of the cofactor, as observed in RutA and EncM.^25,26^ The O_2_ pocket is delineated mostly by nonpolar residues (Ala228, Phe12, Phe56, and Phe246) and by two polar side chains from Asn135 and Thr106 (Figure 5). The O_2_ molecule stays closer to N^5^ (2.72 Å), and to the hydrogen bonded to N^5^ (2.39 Å), than to C^4a^ (3.67 Å). Additionally, the line connecting the proximal oxygen atom of the O_2_ and the N^5^ atom of FMN is almost perpendicular to the longitudinal axis of the flavin (82.9°). This corroborates observations made by other authors in RutA and EncM.^25,26^ In the first mechanistic step (Figure 6), the hydrogen transfer from N^5^ to O_2_ is straightforward (free energy barrier of 2.1 kcal·mol^–1^) and is exergonic with a reaction free energy of −8.1 kcal·mol^–1^, which corroborates the notion that flavin monooxygenases are optimized to bind superoxide to avoid its escape to the cell.^65^ This mechanistic step yields a neutral hydroperoxyl and a negatively charged semiquinone. The analysis of the spin density indicates that in the reactants state, no electron transfer had yet occurred between the flavin and O_2_ as the latter is in the triplet state with a spin density of 1.8. While upon formation of the hydroperoxyl (INT1 of Figure 6), the flavin and the hydroperoxyl have each a spin density of 1, mostly localized at the O_p_ of OOH and the N^5^ of FMN (0.7 and 0.4, respectively). In the TS1 (711.35*i* cm^–1^) structure, a positive partial charge and no spin density in the hydrogen transferred from N^5^ to the O_d_ is observed, which suggests that this step seems better described as a proton-coupled electron transfer.

We optimized INT1 in a triplet and an open-shell singlet (*i.e.*, singlet biradical) state that returned very close energies (less than 0.1 kcal·mol^–1^ apart) and identical geometries, suggesting that the spin flip needed for the superoxide to bind to N^5^ in the following step should easily occur, whereas the closed-shell singlet state stays 28.2 kcal·mol^–1^ above.

The Asn135, which stays at around 2.9 Å from the hydrophobic O_2_ in the reactants, gets closer (2.51 Å) to the more hydrophilic superoxide, suggesting a crucial role in leading O_2_ toward the N^5^ atom. Interestingly, in RutA, mutation of the homologue Asn completely inactivates the enzyme,^25^ highlighting that the Asn135 significantly contributes for the positioning of the dioxygen molecule (and the resulting hydroperoxyl). In the products, a strong hydrogen bond is maintained between the hydroperoxyl and the N^5^ (1.51 Å), while the proximal oxygen stays at 3.04 Å from the N^5^ (Figure 6, INT1).

**Figure 7:**
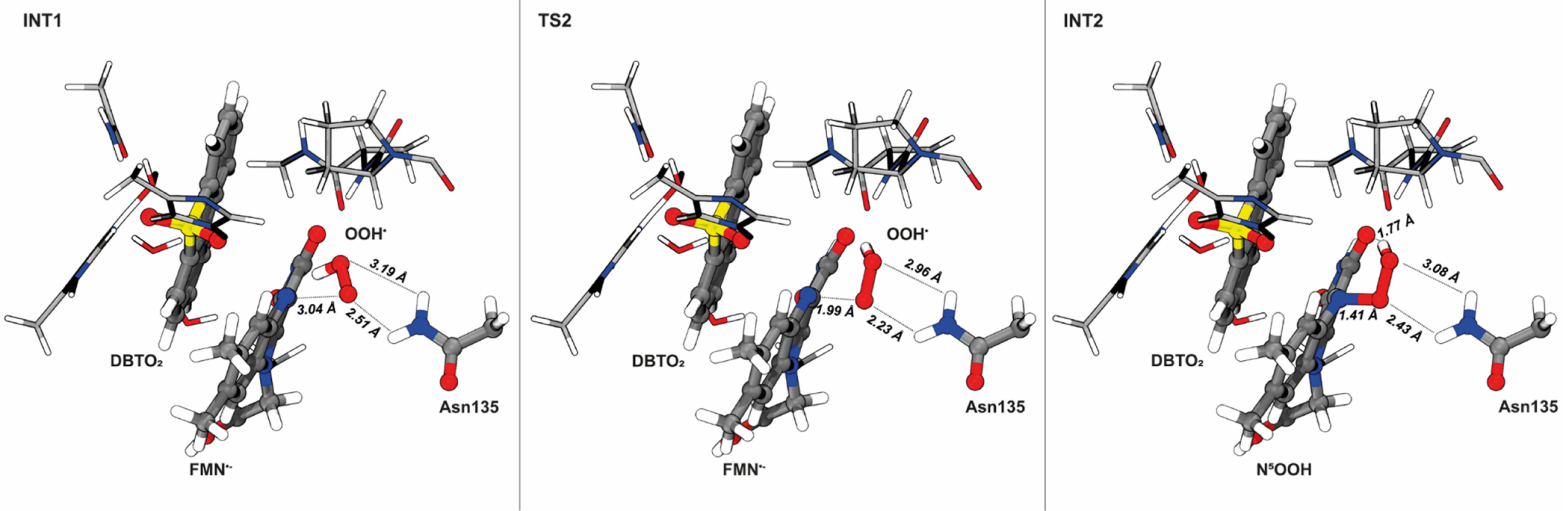
Representation of the stationary points of the second step of the catalytic mechanism of DszA. This step corresponds to the radical coupling between hydroperoxyl and the semiquinone resulting in a N^5^OOH intermediate. The reaction coordinate (defined by the distance between the O_p_ and N^5^) and the lengths of the H-bonds between the hydroperoxyl and Asn135 are highlighted.

The second mechanistic step involves the formation of the N^5^OOH intermediate through a “face-on” radical coupling between the protonated superoxide molecule and the semiquinone. Considering the expected spin transitions, we studied this step in different electronic states, triplet, open-, and closed-shell singlet states, by carrying out vertical spin transitions for the triplet and closed-shell singlet electronic configurations along the minimal energy path of the step calculated in the open-shell singlet spin multiplicity (details in Figure S21). Initially, the system is in a triplet state with the reaction coordinate (distance between the proximal oxygen of OOH and the N^5^) at 3.04 Å. The energies of the triplet and open-shell singlet states remain virtually identical until the reaction coordinate reaches 2.41 Å. From that point on, the triplet state becomes increasingly higher in energy than the open-shell singlet; thus, the spin inversion to an open-shell singlet occurs before the transition state. This is corroborated by a minimal energy crossing point calculation, which predicts that the spin inversion occurs near the INT1 state. In TS2, the O_p_ of the hydroperoxyl is 1.99 Å away from the N^5^ atom and Asn135 gets closer to the O_p_ (2.23 Å) (Figure 7), demonstrating its role in guiding the superoxide toward N^5^ and stabilizing TS2. The TS2 exhibits a sole imaginary frequency (320.25*i* cm^–1^) dominated by the vibration of the O_p_-N^5^ distance accompanied by a slight movement of the side chain of Asn135. After TS2, when the proximal oxygen of the superoxide is closer than 1.87 Å to the N^5^ atom, the closed-shell singlet becomes the most favorable electronic configuration. In the N^5^OOH intermediate, Asn135 stays at 2.43 Å from O_p_ and the O_d_H is donating a hydrogen bond (1.77 Å) to the O^4^ of the isoalloxazine ring. The bond between the N^5^ and the proximal oxygen of OOH is almost perpendicular to the plane of the isoalloxazine (116°) with a length of 1.41 Å. In turn, the O_d_ of the OOH is at 5.07 Å from both the C_α1_ and C_α2_ atoms of DBTO_2_.

This step is rate-limiting with the highest activation free energy of the entire catalytic cycle (17.8 kcal·mol^–1^) and is also an endergonic step with a reaction free energy of 6.8 kcal·mol^–1^ relative to INT1. The activation free energy is overwhelmingly dominated by the enthalpy term, with a −*T*Δ*S* term of just 1.5 kcal·mol^–1^.

**Figure 8:**
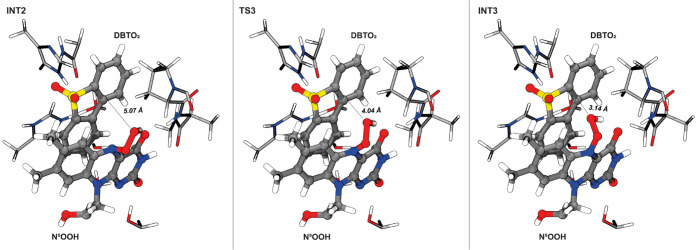
Representation of the stationary points of the third step of the catalytic mechanism of DszA. This step corresponds to the N^5^-inversion that brings the OOH to the cofactor’s *si* face. The distance between the OOH and the C_α2_ atom of DBTO_2_ is highlighted. The improper dihedral angle C^4a^-C^5a^-N^5^–O_p_ oscillates between 126° in INT2 to 162° at TS3 and −150° in INT3.

The catalytic cycle proceeds with an N^5^-inversion in which the OOH goes from the *re* to the *si* face, closer to the substrate. The main reaction coordinate of this step corresponds to the rotation of the improper dihedral angle C^4a^-C^5a^-N^5^–O_p_ from 126° in INT2 to 162° at TS3 and −150° in the product state (INT3). At TS3, we got a sole imaginary frequency (97.39*i* cm^–1^) dominated by the N^5^ inversion that brings OOH from the *re* to the *si* face of the cofactor. In INT3, O_d_ is at 3.14 Å from C_α2_ and at 3.25 Å from C_α1_ of DBTO_2_, which are very favorable distances for the subsequent nucleophilic reaction (Figure 8). The N^5^-hydroperoxyl group maintains the short hydrogen bond with the O^4^ of the cofactor (1.70 Å), while the hydrogen bond between the superoxide and Asn135 increases to 3.38 Å.

The N^5^-inversion exhibited a very low activation free energy of 3.8 kcal·mol^–1^ and was slightly endergonic, with a reaction free energy of 0.6 kcal·mol^–1^.

**Figure 9:**
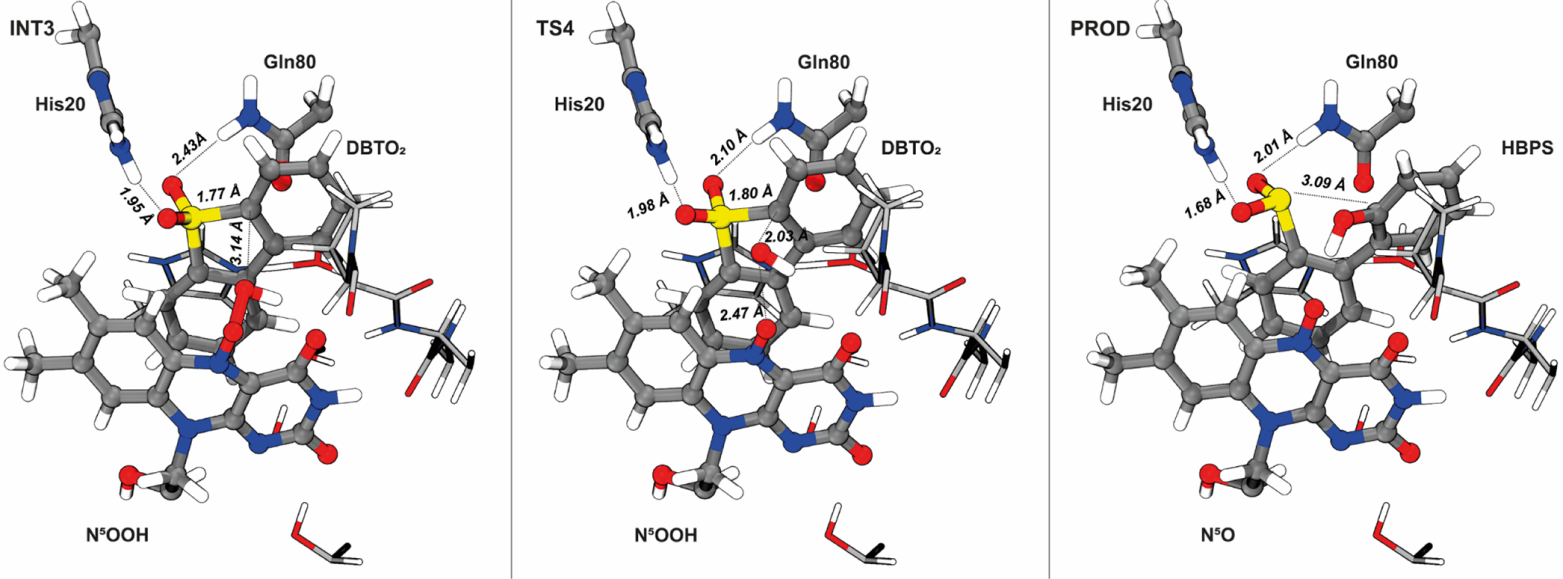
Representation of the stationary points of the fourth step of the catalytic mechanism of DszA. This step corresponds to the OH- transfer from N^5^OOH to DBTO_2_ forming the final product of the mechanism, HBPS. Important distances are highlighted: we calculated an activation free energy of 3.9 kcal·mol^–1^ and a very exergonic reaction free energy of −32.1 kcal·mol^–1^ for this step. The C–S bond breaks during this step and leads to the final product (HBPS) and flavin N5-oxide.

In the last step of the catalytic mechanism, the O_d_ of OOH attacks either the C_α1_ or the C_α2_ of DBTO_2_ to form the final product of the mechanism, HBPS. According to our calculations, the attack on C_α2_ is more favorable. We observed a nonoxidative transfer of a hydroxide molecule from the O_p_ of the cofactor to the C_α2_ of the substrate, which occurred in an asynchronous concerted manner with the cleavage of the C–S bond of the substrate. We did not observe a covalent N^5^OOH-substrate adduct as proposed for RutA,^25^ but on the other hand, our results corroborate the proposal for EncM in which a hydroxide transfer to the substrate is proposed.^26^ A charge analysis points to an heterolytic cleavage of the O_d_–O_p_ bond with the electron density of the bond displacing to the O_d_ atom while N^5^ donates its lone pair to the N^5^–O_p_ bond (Δ*q*_N5_ = 0.29 au; Δ*q*_Op_= −0.26 au). Accordingly, the length of the N^5^–O_p_ bond decreases from 1.31 Å at INT3 to 1.24 Å in the final product state. At TS4, the distance between O_d_ and O_p_ is 2.47 Å and the distance between O_d_ and C_α2_ of DBTO_2_ is 2.03 Å. TS4 is characterized by a sole imaginary frequency (205.64*i* cm^–1^) dominated by the stretching/compression of the O_d_–O_p_ and O_d_–C_α2_ distances. Once past TS4, the length of the C_α2_–S bond on the substrate increases from 1.80 to 3.09 Å at the product state to hold the final HBPS reaction product (Figure 9). A charge analysis confirms the formation of a negatively charged sulfinate group (Δ*q*_SO2_ = −0.37 au), which is accompanied by the shortening of the hydrogen bonds between the sulfinate group and the His20 and Gln80 (from 1.95 to 1.68 Å and from 2.43 to 2.01 Å, respectively).

### Thermochemical Profile of the Reaction Mechanism

**Figure 10:**
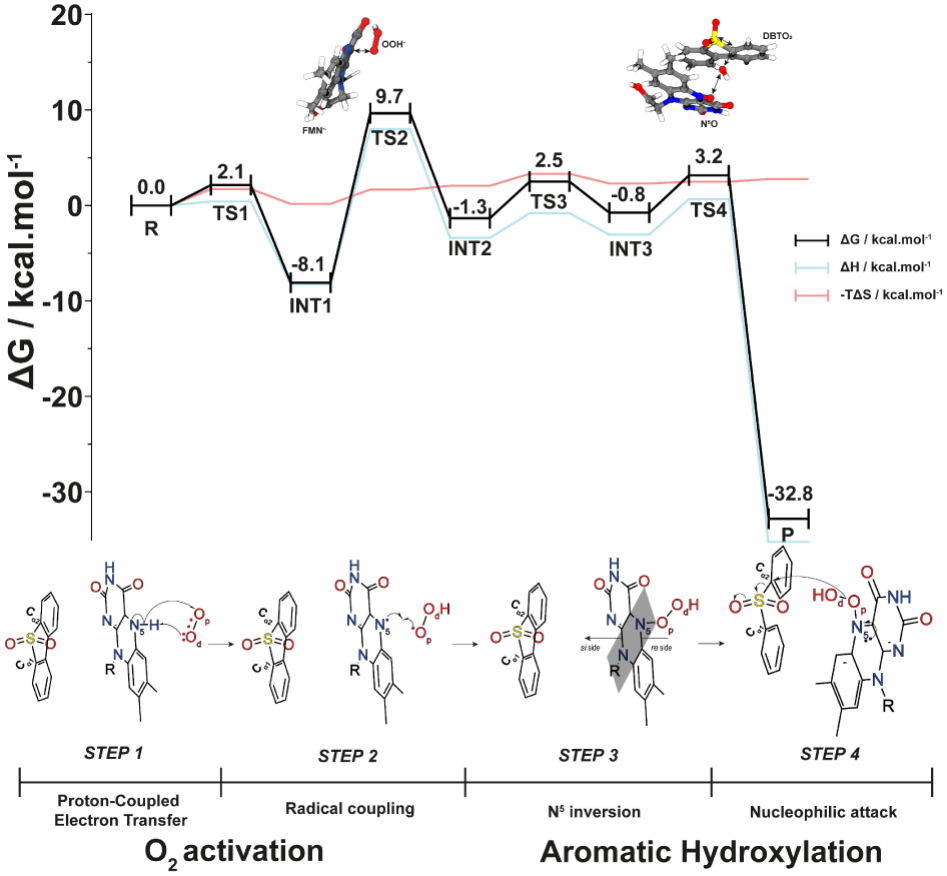
Free energy profile obtained for the catalytic mechanism of DszA calculated at the PWPB95-D3BJ/def2-TZVPP:ff10//PBE0-D3BJ/def2-SVP:ff10 level of theory. The rate-limiting step corresponds to the radical coupling between the hydroperoxyl molecule and the semiquinone with an activation free energy of 17.8 kcal·mol^–1^. Simplified structural representations of the rate-limiting steps of each reaction stage, radical coupling—at the oxygen activation stage—and nucleophilic attack—at the aromatic hydroxylation stage—are shown above the energy profile, and 2D representations of each mechanistic step are shown at the bottom of the figure. The nomenclature used in the text for the atoms that are important for catalysis is represented.

The overall free energy profile is shown in Figure 10. The rate-limiting step of the catalytic mechanism has an energy barrier of 17.8 kcal·mol^–1^ and corresponds to the binding of the hydroperoxyl molecule to the N^5^ atom of FMN with concomitant spin inversion, bringing the system from a triplet to a singlet state. This value compares well with experimental data that indicates an activation free energy of 18.6 kcal·mol^–1^.^62^ Even though the formation of the N^5^OOH intermediate is endergonic, both the inversion of the N^5^–OOH from the *re* to the *si* face of FMN and the nucleophilic attack of the O_d_H as an hydroxide to the C_α1_/C_α2_ require a total free energy barrier of 4.5 kcal·mol^–1^, which is lower than the required to revert N^5^OOH formation (11.0 kcal·mol^–1^) and thus should not compromise the catalytic rate of the enzyme at this stage. In summary, our results support the formation of a flavin N^5^OOH intermediate at the *re* side of the cofactor in the DszA catalytic cycle, in agreement with works on RutA and EncM.

As to why the N^5^-pathway is favored over the C^4a^-pathway, our results suggest that the O_2_ pocket at the *re* face of the flavin cofactor (represented in Figure 5) should be instrumental for the specificity of N^5^OOH formation over C^4a^OOH, which in turn promotes the hydroxylation of DBT-sulfone with a high catalytic rate. In fact, the cofactor seems to play a determining role in the catalysis of this reaction by DszA, as an analysis of the DBT-sulfone hydroxylation step for both the C^4a^-pathway (Figure S20) and the N^5^-pathway (Step 4 in Figure 10) with the activation strain model of Vermeeren^66^ indicates that the strain on the cofactor (35 kcal·mol^–1^ vs 7 kcal·mol^–1^) is the dominant contribution for the difference in this reaction barrier (detailed analysis can be found in Supporting Information). As such, as previously hypothesized by Su et al. for the orthogonal enzyme BdsA, DszA residues do not intervene in the chemical reactions taking place in the enzyme but instead provide the appropriate environment for N^5^OOH formation and consequent DBT-sulfone hydroxylation.

Zhang and colleagues^44^ propose that the lack of a proton donor on flavin enzymes that promote the formation of N^5^OOH (specifically EncM and RutA) is also a determining factor. C^4a^OOH forming enzymes contain a conserved His residue in the active site that mediates proton-coupled electron transfer to form the hydroperoxyl radical. This, however, may not be the case for DszA since His316, located in the active site on the *si* side of the cofactor, could replicate the role of the conserved His of C^4a^OOH forming enzymes. Thus, our results point to the existence of an O_2_ pocket near the N^5^ atom at *re* side of cofactor as the determining factor in controlling N^5^OOH *vs* C^4a^OOH formation.

## Conclusions

In this work, we used computational methods to explore the catalytic cycle of the FMN monooxygenase DszA. We tested two mechanistic hypotheses that vary with the covalent flavin-oxygen adduct formed. The more common one for equivalent reactions involves the formation of a C^4a^OOH intermediate and the other recently proposed one involves the formation of an N^5^OOH intermediate. The latter was based on the observation of an N^5^-oxide species in some flavin monooxygenases that cleave carbon-hetero bonds, such as DszA,^24^ RutA,^27^ HcbA,^67^ and EncM.^68^

The pathway through C^4a^OOH is unviable due to the high barrier (above 30 kcal·mol^–1^) for the transfer of OH to DBTO_2_ and the breaking of the C–S bond. Such a high barrier is likely due to the unavailability of redox species within the enzyme other than the FMN and dioxygen themselves, which render it unlikely for an OH transfer from C^4a^OOH to the DBTO_2_ substrate of DszA.

In turn, the pathway through N^5^OOH is energetically favorable. First, we confirmed the existence of a hydrophobic O_2_ pocket at the *re* side of the cofactor, stabilizing the dioxygen molecule near the N^5^ atom of FMNH. After the hydrogen transfer from N^5^ to O_2_, the radical coupling between the hydroperoxyl molecule and the semiquinone occurs concerted with the spin inversion from triplet to singlet state, and Asn135 seems responsible for guiding OOH toward N^5^. These structural features of DszA help explain why the activation of O_2_ occurs at the *re* side of the cofactor, which steers the formation of N^5^OOH instead of the more common “face-on” approach of O_2_ to C^4a^ at the *si* side. An N^5^–OOH inversion from the *re* to the *si* face of the cofactor quickly follows, along with a nucleophilic attack by an hydroxide species to the C–S bond of DBTO_2_, forming the HBPS product in a highly exergonic reaction. Contrary to the C^4a^OOH intermediate, the N^5^OOH intermediate uses the N^5^ as a reducing agent to reduce the proximal oxygen, forming an N^5^-oxide, and allows the release of a hydroxide nucleophile.

The formation of the N^5^OOH intermediate is the rate-limiting step of the catalytic reaction with a barrier of 17.8 kcal·mol^–1^. This does not corroborate with results obtained in small model DFT calculations by Matthews^25^ and Batcha,^26^ in which bond cleavage exhibited the highest barrier, but agree with recent insights on the reaction mechanisms of EncM and RutA determined by QM/MM calculations in which the authors demonstrated that the mechanistic step involving the spin inversion and radical coupling exhibits the highest energy barrier.^44^ Our energy profile also compares well with experimental k_cat_, which suggests an activation free energy of 18.6 kcal·mol^–1^.

The recent proposal of the involvement of the N^5^ position in flavoenzyme catalyzed reactions further highlights the remarkable versatility of flavin-based cofactors. Our results corroborate that the existence of a hydrophobic O_2_ pocket at the *re*-side of FMN in flavin containing enzymes, which catalyze oxygenase reactions, leads to the formation of a N^5^OOH intermediate instead of the better known C^4a^OOH. This further highlights the importance of the N^5^ position in flavin-catalyzed oxygenase reactions, as the one conducted by DszA, as observed by other authors,^69^ and in particular for flavin-mediated C–S bond cleavage as also demonstrated by Hazra and Begley,^70^ which recently elucidated a novel catalytic reaction of the flavin monooxygenase CmoJ that also involves N^5^-hydroperoxyl formation, and by Wu and Chen, which proposed that C–S bond cleavage catalyzed by MsuD requires a N5OO^–^ intermediate.^71^

We expect future work to try to elucidate which type of flavin-catalyzed reactions, in addition to monooxygenase reactions that cleave carbon-hetero bonds, are more prone to happen with the participation of the N^5^ or the C^4a^ position and which other structural features may influence the likelihood of each pathway. Also, the revisitation of previous mechanistic studies on flavin enzymes that were dogmatically proposed as going through the C^4a^ pathway may be motivated.

## Data Availability

The coordinates for all stationary states in the suggested mechanism; ORCA Inputs and outputs of the computations that provided the data presented in the manuscript for the proposed mechanism and the necessary ORCA parameter files are available on a Zenodo repository at zenodo.org/doi/10.5281/zenodo.10696957.

## Supporting Information Available

Data regarding the construction of the computational model of the enzyme; discussion of the molecular docking carried out to obtain the enzyme:substrate complex; results and discussion of the C^4a^OOH pathway; analysis of the electronic configuration of step 2 of the N^5^OOH pathway; discussion on the impact of different DFT functionals on the energy profile; results and discussions on the energy decomposition analysis carried out to study the contributions to the energy profile (PDF)
