# Supplementary material for: DszA Catalyzes C–S Bond Cleavage through N5–Hydroperoxyl Formation
Source: J Chem Inf Model. 2024 Apr 29;64(10):4218–30. doi: 10.1021/acs.jcim.4c00301 (PMC11134501; doi:10.1021/acs.jcim.4c00301)
Supplement: Supplementary file 1 — ci4c00301_si_001.pdf [file ci4c00301_si_001.pdf]

Supporting Information

DSZA CATALYZES C-S BOND CLEAVAGE THROUGH N5-HYDROPEROXYL FORMATION

Pedro Ferreira[a], Rui P. P. Neves[a], Filipa P. Miranda[a], Ana V. Cunha[b], Remco W. A. Havenith[c], Maria J. Ramos[a], and Pedro A. Fernandes[a,*]

[a]LAQV,REQUIMTE, Departamento de Química e Bioquímica, Faculdade de Ciências, Universidade do Porto, Rua do Campo Alegre, s/n, 4169-007 Porto, Portugal

[b]Department of Chemistry of the University of Antwerp, Groenenborgerlaan 171, 2020 Antwerp, Belgium

[c] Stratingh Institute for Chemistry and Zernike Institute for Advanced Materials, University of Groningen, Nijenborgh 4, 9747 AG Groningen, The Netherlands, and Ghent Quantum Chemistry Group, Department of Chemistry, Ghent University, Krijgslaan 281 (S3), B-9000 Gent, Belgium

E-mail: pafernan@fc.up.pt

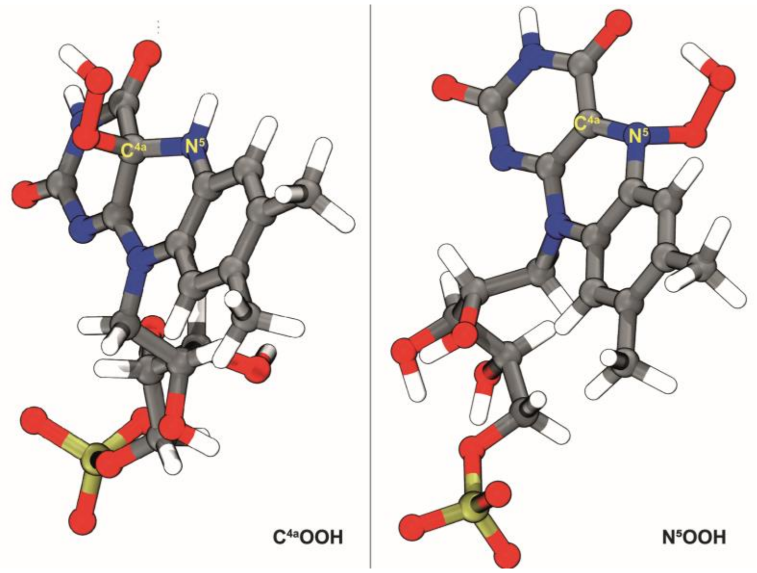

Figure SI 1. Representation of the two forms of flavin hydroperoxide modeled to study the catalytic mechanism of DszA. On the left it is represented the C4aOOH form, with the OOH at the si face of the cofactor. On the right it is represented the N5OOH form with the OOH at the re face of the cofactor. The location of the C4a and N5 atoms are marked in yellow.

Modeling of DszA:FMN

The DszA model was obtained as a tetramer from the Swiss-Model webserver, after a BdsA homolog with a sequence identity of 79.28%, with a QMEAN value of -0.62 and a GMQE value of -0.62. The Ramachandran plot the model demonstrates that its secondary structure is comparable to the ones commonly found in nature, with a percentage of outliers of only 0.40% (Val371 of chain A, B and D, Gln338 of chain D, Ser355 of chain A and B, and His354 of chain D). As for the comparison of the model to other naturally existing structures, the QMean Z-Score is less than one, thus falling into the most populated structure area, demonstrating its likelihood of existing in nature, although there are few tetramer structures available for statistical validation. To further demonstrate that the attained model is similar to other structures existent in nature, each chain was compared to experimentally determined structures in ProSA-web, [1] further supporting the obtained homology model. These good quality results indicate that the obtained model should be of high quality, thus representing a reliable DszA structure. Summary of the results follows in **Figure SI 2**.

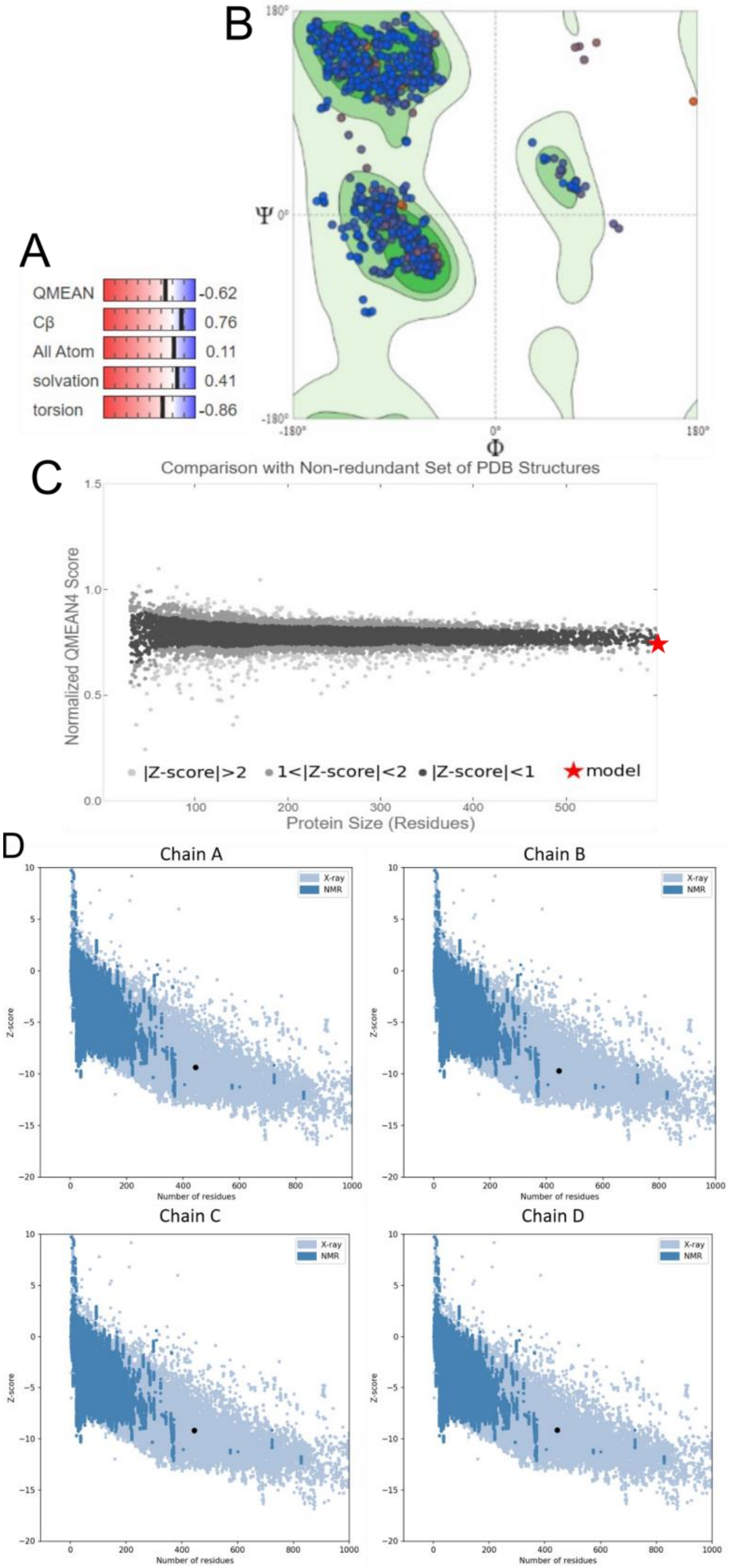

Figure SI 2. A) Quality values for DszA model, where darker blue colors corresponds to better quality; B) Ramachandran plot for DszA model; C) Comparison of DszA model with non-redundant set of PDB Structures; D) Comparison of chain A, B, C, and D of DszA model with set of X-ray and NMR structures (from ProSA-web).

The superimposition of the model DszA with its template, BdsA, exhibited an RMSd value of 0.134 Å over all backbone atoms, which indicates that the structures are very similar. The residues at the active center of BdsA that, according to Su *et al.*, are essential residues for BdsA activity are Phe12, Phe56, Phe246, His20, His316, Val248 and Val372. Observing the active sites from both systems (seen in **Figure SI 3**), it is noticeable that the position and orientation of the mentioned residues on the active site of the modelled DszA and of BdsA are aligned and indeed very similar. Nonetheless, there is a residue deletion, as Val372 of BdsA is correspondent to Val371 of the model, and Val248 is substituted by Leu248. However, this latter substitution should not be a problem as both residues have similar sizes and hydrophobic characteristics. Thus, since BdsA and DszA are similar, these residues are also probably essential for DszA activity. Nonetheless, the vast resemblances between the model and FMN-bounded BdsA, further indicates that the attained model is in fact trustworthy and of quality.

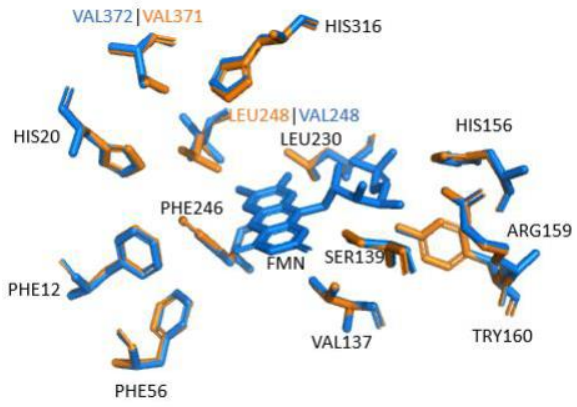

Figure SI 3. Active center of BdsA (blue) vs active center of model DszA (orange).

Due to their structural similarities, the molecular modeling of the oxidized FMN from BdsA to DszA should be a fairly low-risk process. The DBT-sulfone substrate should then bind at the *si* face of the FMN cofactor, likely establishing hydrogen bonds with the surrounding residues, possibly with the FMN cofactor, His316 and His20.

Based on the comparison between similar active homodimers, namely nitrilotriacetate monooxygenase (PDB ID: 3SDO), alkane monooxygenase (PDB ID: 3B9N) and riboflavin lyase (PDB IDs: 5W4Y; 5W4Z; 5W48), from which the RMSd variations

among backbone atoms varied between 1.6 and 1.8 Å, DszA was finally modelled as a homodimer including chains A and B, in complex with FMN, in a total 6940 atoms.

The DszA:FMN complex was then modelled as DszA:$C^{4a}OOH$, which corresponds to the oxygen-activated form of the FMN cofactor in agreement with most literature, and underwent MD simulations. The RMSd of a 50 ns production showed that the RMSd for chain A was slightly lower than for chain B, both for the backbone and the active site atoms (**Figure SI 4**). Nevertheless, a visual analysis indicated that the differences in the RMSd of backbone atoms were mostly due to the dynamics of a terminal loop with no relevance for the activity of DszA.

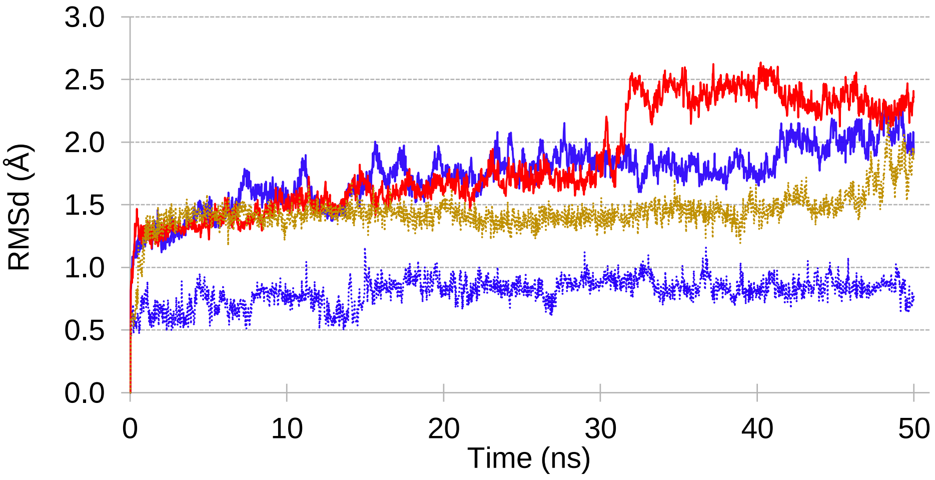

Figure SI 4. RMSd values for backbone (solid lines) and active site heavy atoms (dotted lines) in chain A and B, throughout the 50 ns NPT production. Chain A is colored blue and chain B is colored red.

Su *et al.* describes that the BdsA cofactor, FMN, is held by hydrogen bonds with residues Asp59, Ser139, His156, Arg159, Tyr160 and Leu230, and by a hydrophobic interaction with Val137 (residues conserved in DszA). An analysis of the hydrogen bonds at the active site of chain A and B during a total 100 ns MD simulations, indicates that the hydrogen bonds established by the backbone of Asp59 and His156 are the most long-lasting, followed by those by Ser139. The remaining residues interchange with other neighboring residues to conserve the hydrogen bond network around $C^{4a}OOH$. A representative structure was obtained by performing a geometric clustering using the RMSd of the heavy atoms of each active site as a metric, is performed for a 100 ns *NPT* MD simulation. After superimposing the active site of the resulting dominant cluster with

that of BdsA, we obtained a comparative RMSd of 0.691 and 0.546 Å for the heavy atoms of active sites A and B, respectively (**Figure SI 5**).

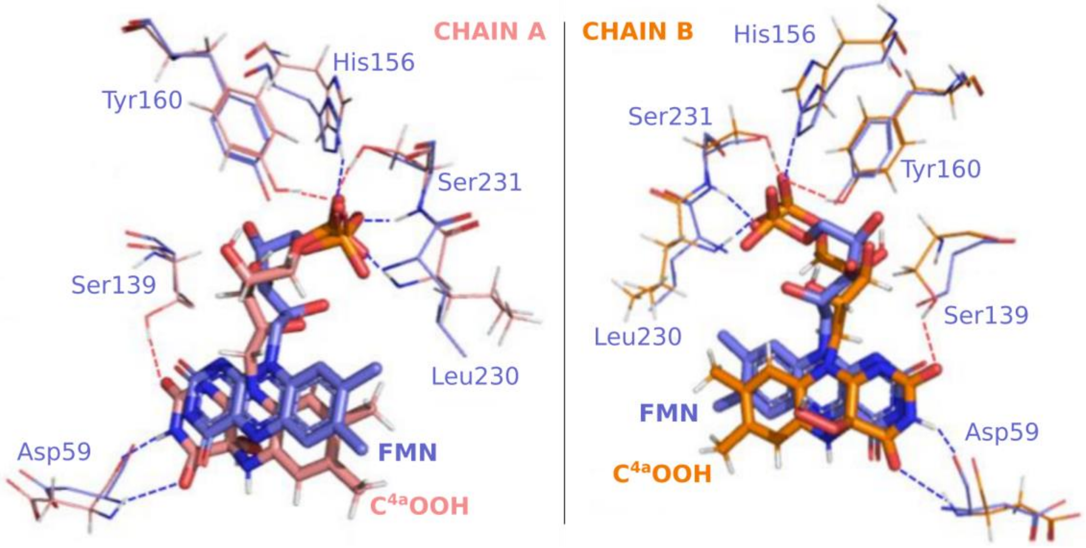

Figure SI 5. Superimposition of the active sites A and B from the dominant cluster of DszA (colored in pink for chain A and in orange for chain B), with those of BdsA (colored in purple). Not all residues that establish hydrogen bonds with the cofactor are depicted for clarity. Dashed blue and red lines highlight hydrogen bonds from nitrogen or oxygen donor atoms, respectively.

Through all the steps, the system has shown to be of quality as the initial model obtained from homology modeling should be reliable and upon energy minimizations and MD simulations, it became equilibrated and maintained most of the interactions thought to be important for cofactor binding. Moreover, comparison with the X-ray structure of its homolog, BdsA, shows no major structural modifications at the active site, which further renders our model as viable to proceed for substrate modelling.

Modeling DBT-sulfone binding

Docking of DBT-sulfone to the active site of DszA was conducted on both DszA:C^{4a}OOH and DszA:N^5OOH complexes previously subject to an energy minimization.

The docking of DBT-sulfone was performed with AutoDock Vina, using the Lamarckian genetic algorithm to explore a search space encompassing the FMN cofactor and the residues composing the *si* face of the active site of DszA (Ser10, Thr15, His16, Gln76, Asn137, Leu244, Thr308, Ser311, His312, Val367), and the built-in AutoDock Vina scoring function. Selection of the final poses to follow for validation with MD simulations included additional criteria motivated by the available mechanistic hypotheses for DszA: distance between 3-5 Å between the distal oxygen (O_d) of the cofactor and either the Cα1 or Cα2 of DBT-sulfone, lower accessibility to solvent by the DBT-sulfone, and number of poses binding a similar region.

While one solution obeying all criteria was found for the DszA:N^5OOH, we struggled to obtain solutions in which the O_d-Cα1 or O_d-Cα2 distances were within the defined 3-5 Å criteria for the DszA:C^{4a}OOH complex. Hence, we carried an alternative protocol using the GOLD software using the genetic algorithm and a search space of 10 Å within the C^{4a}OOH, in which a biasing harmonic restraint of 5 $N \cdot m^{-1}$ between the Od and the topologically equivalent Cα1 and Cα2 of DBT-sulfone was considering during the scoring step, where the CHEMPLP function was used. The most favorable poses of both protocols are shown in **Figure SI 6**.

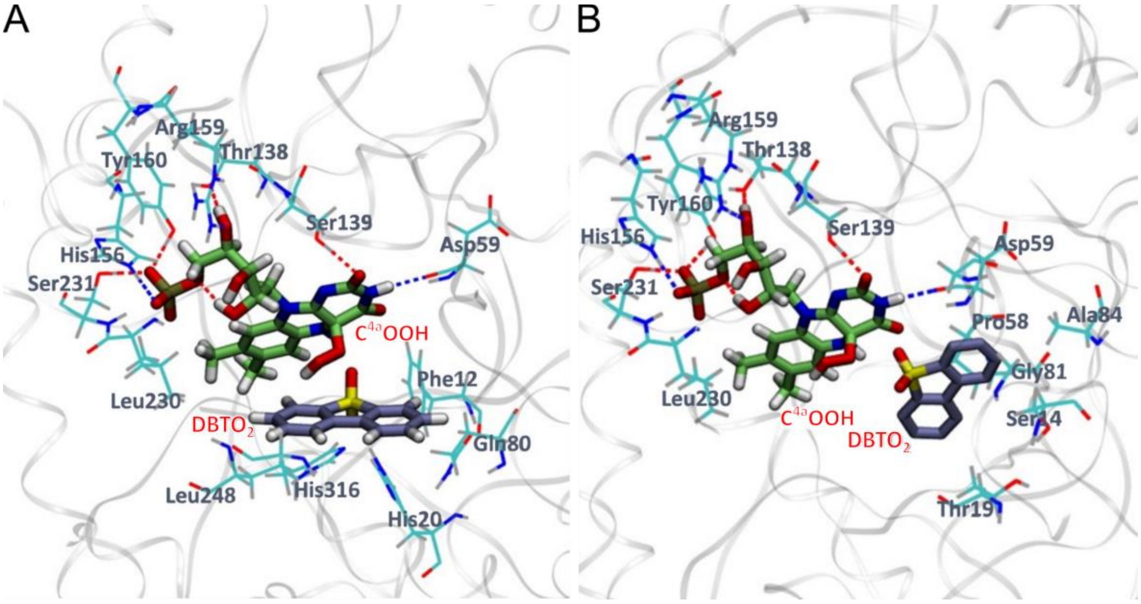

Figure SI 6. Most promising poses for DBT-sulfone for the DszA:C^{4a}OOH complex, using the A. Gold with a bias on the Od-Cα1/2 distance during the scoring step; B. AutoDock Vina. Noticeable polar interactions are represented with dashed lines.

Interestingly, the pose in **Figure SI 6A** is observed in both softwares, although it scores higher in the GOLD software. In addition, it has a favorable O_d-Cα1 distance (3.60 Å) and it is buried into the active site of DszA. As for the pose in **Figure SI 6B**, it also exhibits a favorable O_d-Cα1 distance (3.53 Å), it was one of the best solutions from AutoDock Vina and it is also buried at the active site. Both systems proceeded for cMD simulations to assess the behavior the obtained poses in the active site of DszA. After 100 ns of cMD simulations, the RMSd over the backbone atoms of the DszA homodimer reached a plateau at around 2 Å, which indicates that no major changes occur in DszA once $DBTO_2$ bound the active site (**Figure SI 7**).

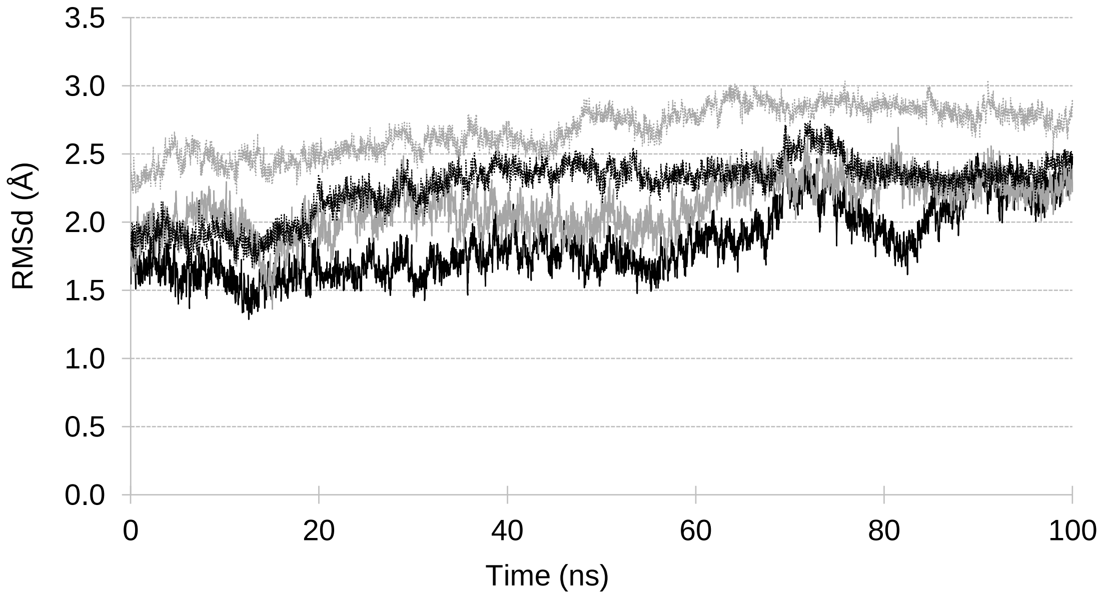

Figure SI 7. RMSd values for backbone (solid lines) and active site heavy atoms (dotted lines) for the DszA:C^{4a}OOH:$DBTO_2$ models built from the $DBTO_2$ poses in **Figure SI 6** (A in black and B in gray) , throughout the 100 ns NPT production.

On a closer look at the RMSd over the heavy atoms of the C^{4a}OOH cofactor and the $DBTO_2$ substrate (**Figure SI 8**), it becomes clear that lower RMSd values are registered whenever the pose obtained from GOLD software is simulated (**Figure SI 6**A).

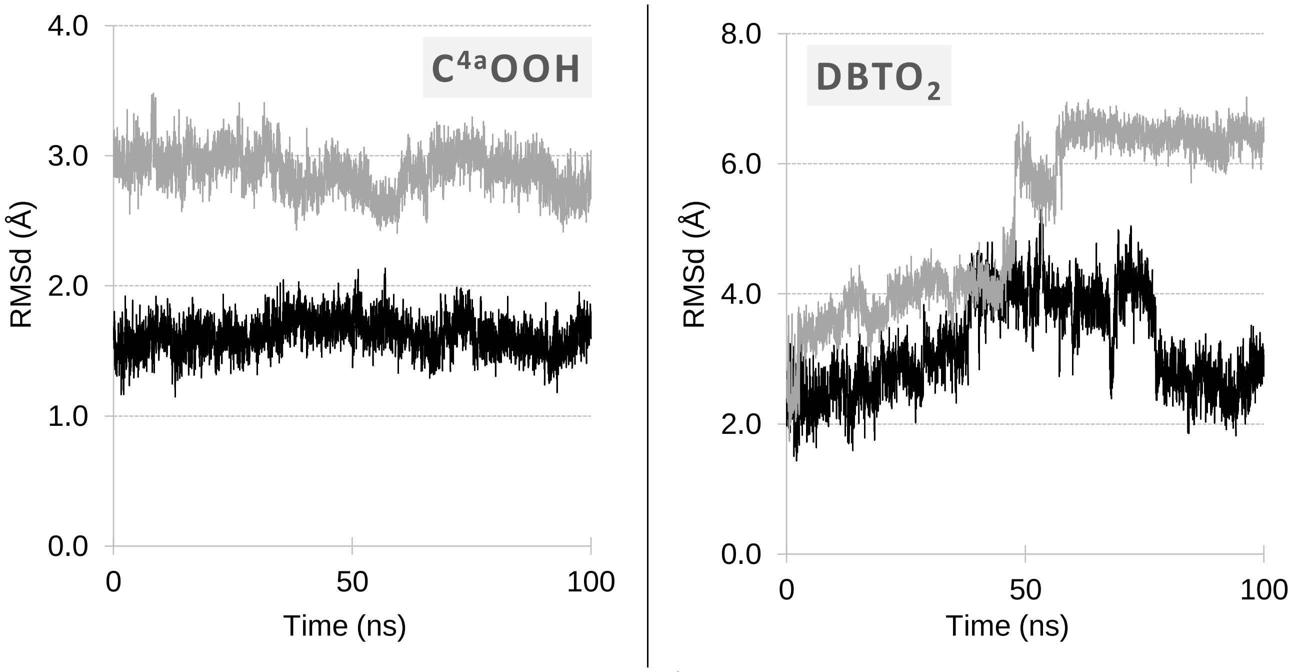

Figure SI 8. RMSd values for heavy atoms of the C^{4a}OOH cofactor and the $DBTO_2$ substrate for the DszA:C^{4a}OOH:$DBTO_2$ models built from the $DBTO_2$ poses in **Figure SI 6** (A in black and B in gray) , throughout the 100 ns NPT production.

These results are further supported by a contact analysis that revealed that most of the conserved interactions between DszA and C^{4a}OOH (Asp59, Thr138, Tyr160, Ser231, His156 and Arg159) are lost at least for one of the monomers whenever the $DBTO_2$ binding pose of **Figure SI 6**B is simulated. When it comes to the binding pose of **Figure SI 6**A, C^{4a}OOH maintains most of the conserved interactions with DszA and $DBTO_2$ exhibits one dominant binding pose anchored by short hydrogen bonds with the N^5 of C^{4a}OOH and the NeH of His20 (Figure SI 9, in line with previous observations)[2].

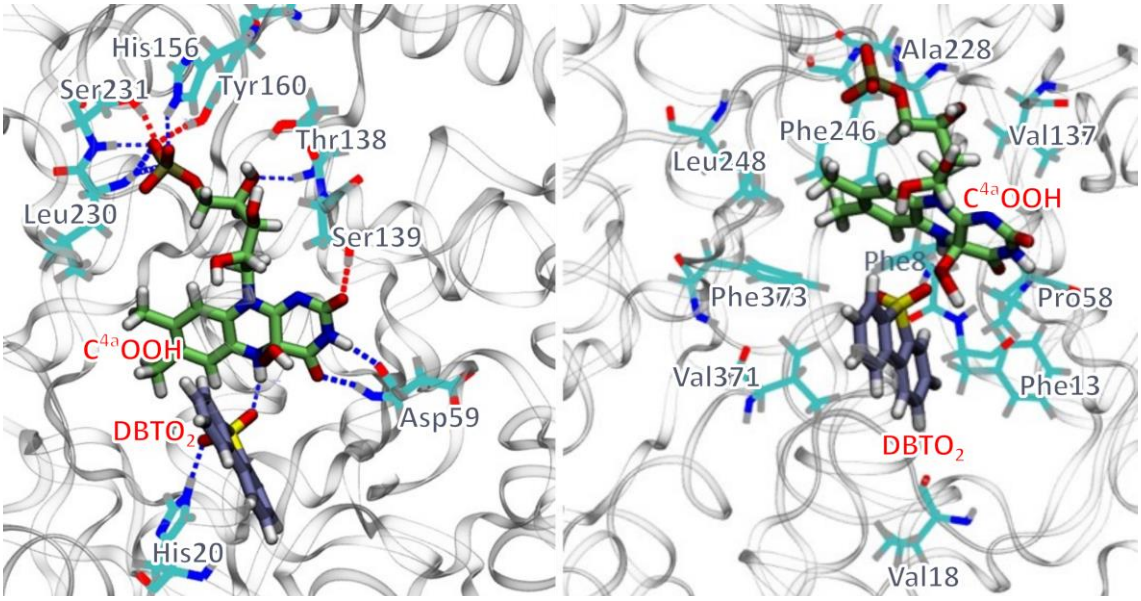

Figure SI 9. Representation of the most relevant polar (on the left) and hydrophobic (on the right) contacts for the best representative pose of the DszA:C^{4a}OOH:$DBTO_2$ complex.

Altogether, the results suggest that the DBTO2 binding pose in **Figure SI 6**A should be more stable, while still supporting the mechanistic hypothesis standing for the reaction mechanism of DszA in complex with a C^{4a}OOH cofactor.

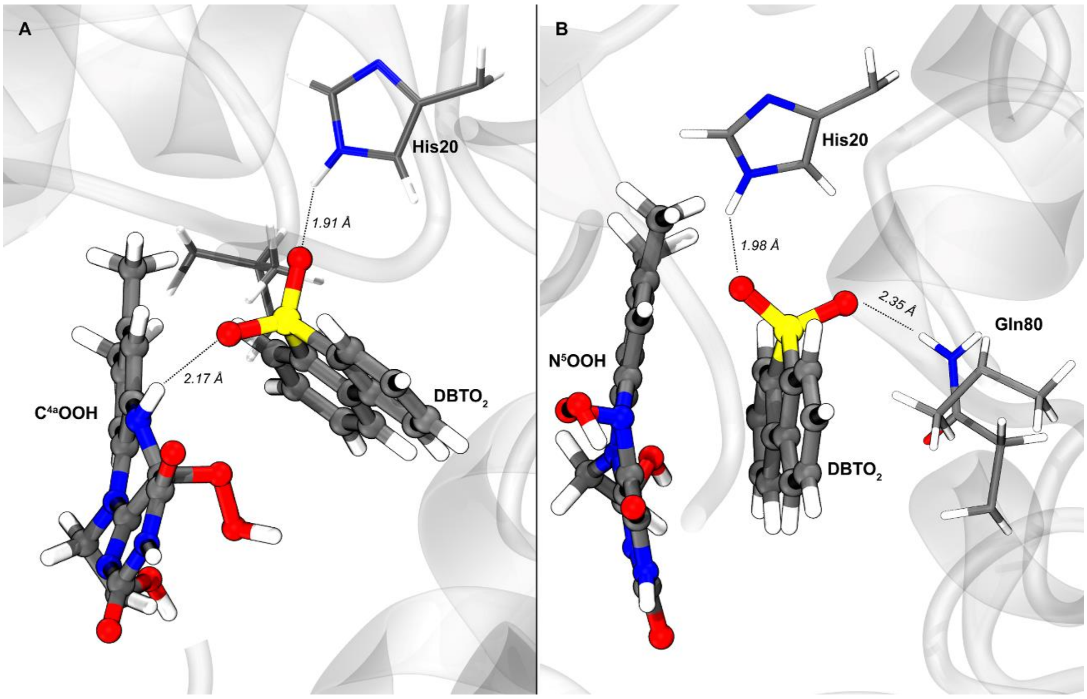

Figure SI 10. Representation of the minimized structure of DszA in complex with the best docking poses of $DBTO_2$ considering the C^{4a}OOH (**A**) and the N^5OOH (**B**) cofactor. H-bond distances between the substrate and the active site are shown.

The $C^{4a}OOH$ vs N^5OOH model

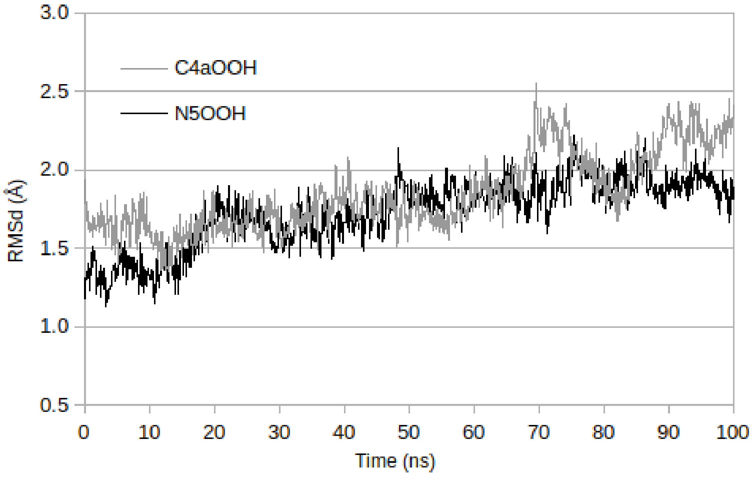

Figure SI 11. RMSd for the backbone atoms of the DszA dimer for both the DszA:C^{4a}OOH:$DBTO_2$ and the DszA:N^5OOH:$DBTO_2$ complexes.

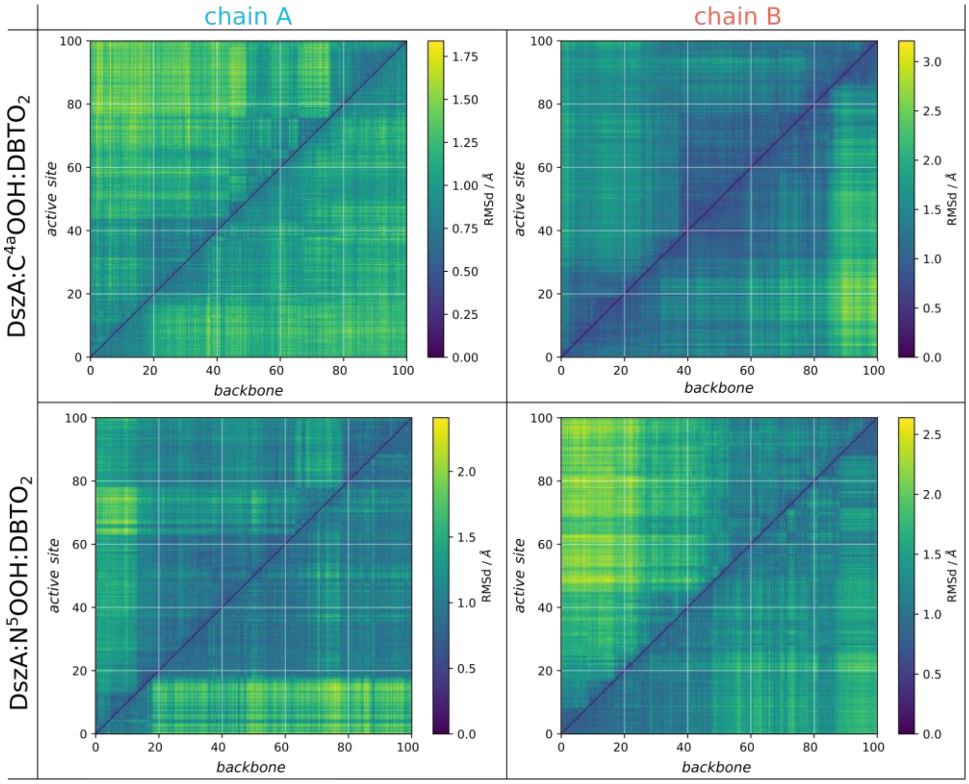

Figure SI 12. 2D-RMSd plots of the backbone (lower triangle) and active site (upper triangle) heavy atoms for chains A and B of the DszA:C^{4a}OOH:$DBTO_2$ and the DszA:N^5OOH:$DBTO_2$ complexes.

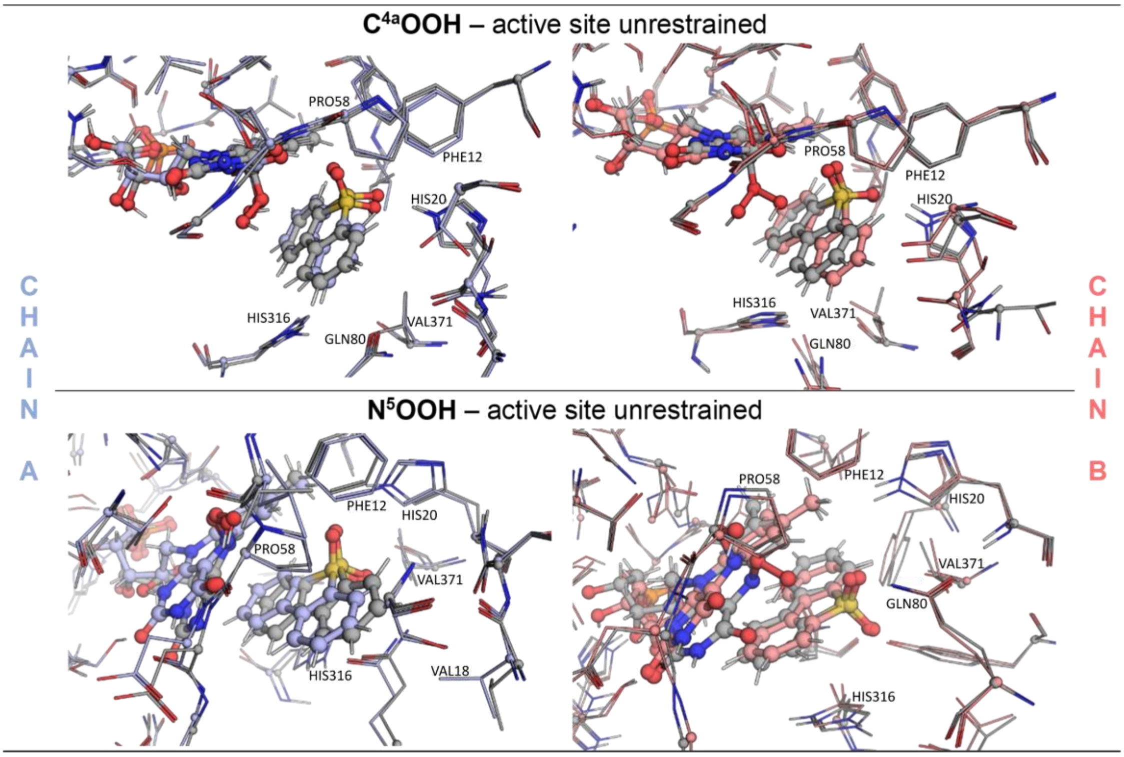

Figure SI 13. Active site representation of the centroid structure for each of the chains A and B in the DszA:C^{4a}OOH:DBTO$_2$ (top) and the DszA:N^{5}OOH:DBTO$_2$ (bottom) complexes, along the 10 ns molecular dynamics simulations where DszA residues beyond the active site were kept restrained.. The minimized structure used as reference was colored in light blue (chain A) and light pink (chain B), and the representative structures from the clustering are colord in gray; the modelled FMN cofactor and the DBTO$_2$ substrate are shown in ball-and-stick representation. Clustering was perfomed over the RMSd of the heavy atoms in the selection defined as the active site, with the GROMOS algorithm and a 1.0 Å cutoff. Native residues suggested by the docking poses are labelled.

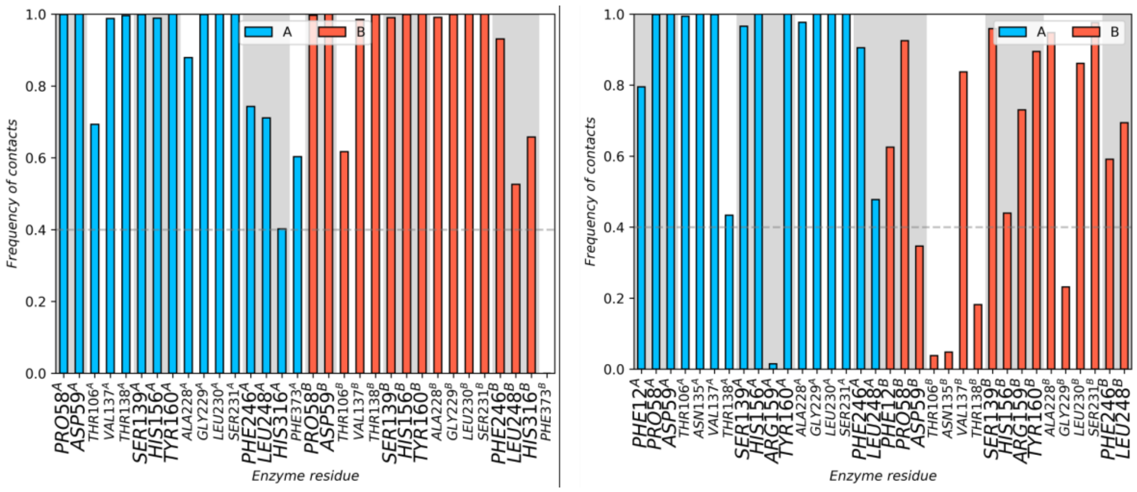

Figure SI 14. Frequency of contacts between the heavy atoms of C^{4a}OOH (left) and N^{5}OOH (right) and the DszA residues within 4 Å, for both chain A (in blue) and B (in red), along the 100 ns molecular dynamics simulations.

Table SI 1. Summary of the clustering results over the RMSd of the heavy atoms of the active site selection along the 100 ns molecular dynamics simulations, using the GROMOS algorithm and a 1.4 Å cutoff. Each entry shows the RMSd of heavy atoms of the modelled FMN cofactor and the $DBTO_2$ substrate in the representative structure of each cluster in relation to the minimized structure, as well as the relative occupation of each cluster. Entries colored in light green and yellow correspond to representative structures with RMSd below 2 Å and above 3 Å, respectively, for both the modelled FMN cofactor and the $DBTO_2$ substrate.

	**$C^{4a}OOH$ | $DBTO_2$ (occupancy)**		**N^5OOH | $DBTO_2$ (occupancy)**	
	Chain A	*Chain B*	*Chain A*	*Chain B*
1	**1.6 | 3.0 (100%)**	1.8 | 3.7 (80%)	**1.4 | 1.9 (99.1%)**	2.1 | 1.6 (68.4%)
2	-	1.4 | 2.2 (15%)	1.6 | 5.0 (0.6%)	1.2 | 0.7 (25.2%)
3	-	1.4 | 2.3 (4.6%)	1.5 | 3.3 (0.4%)	2.2 | 2.1 (4.6%)
4	-	2.1 | 4.5 (0.4%)	-	2.4 | 1.2 (1.3%)
5	-	-	-	1.2 | 0.9 (0.6%)

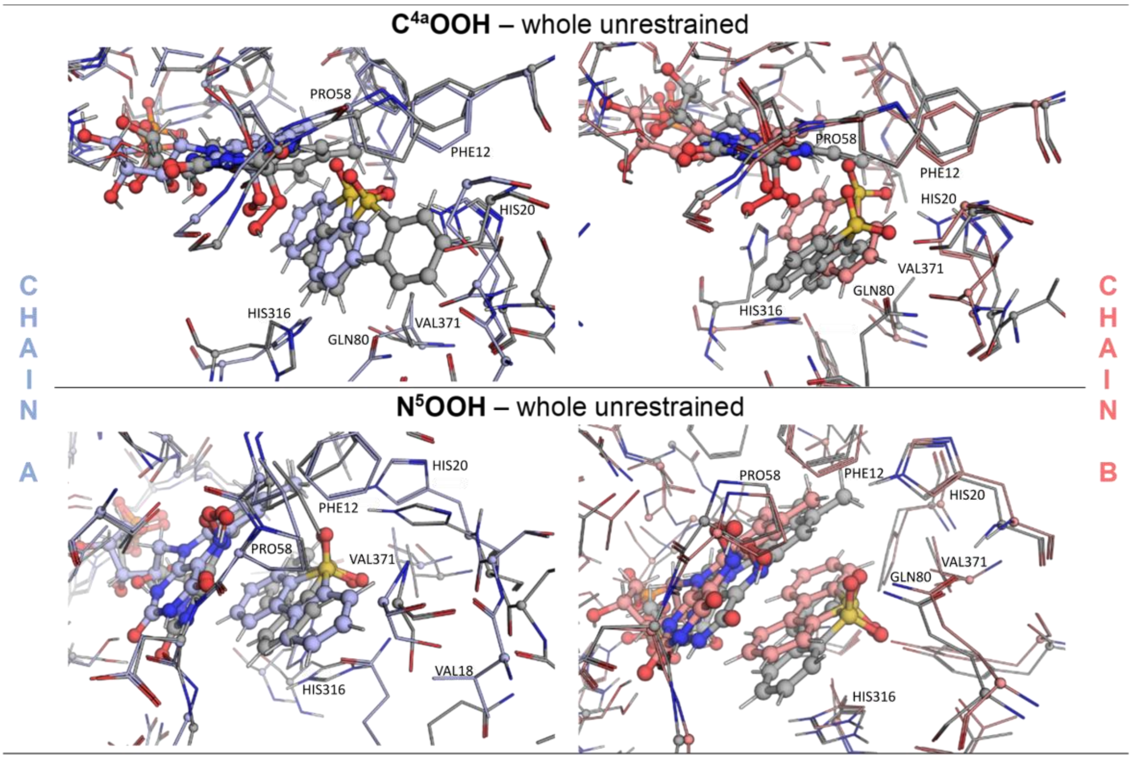

Figure SI 15. Active site representation of the centroid structure for each of the chains A and B in the DszA:$C^{4a}OOH$:$DBTO_2$ (top) and the DszA:N^5OOH:$DBTO_2$ (bottom) complexes, along the unrestrained 100 ns molecular dynamics simulations. The minimized structure used as reference was colored in light blue (chain A) and light pink (chain B), and the representative structures from the clustering are colord in gray; the modelled FMN cofactor and the $DBTO_2$ substrate are shown in ball-and-stick representation. Clustering was perfomed over the RMSd of the heavy atoms in the selection defined as the active site, with the GROMOS algorithm and a 1.4 Å cutoff. Native residues suggested by the docking poses are labelled.

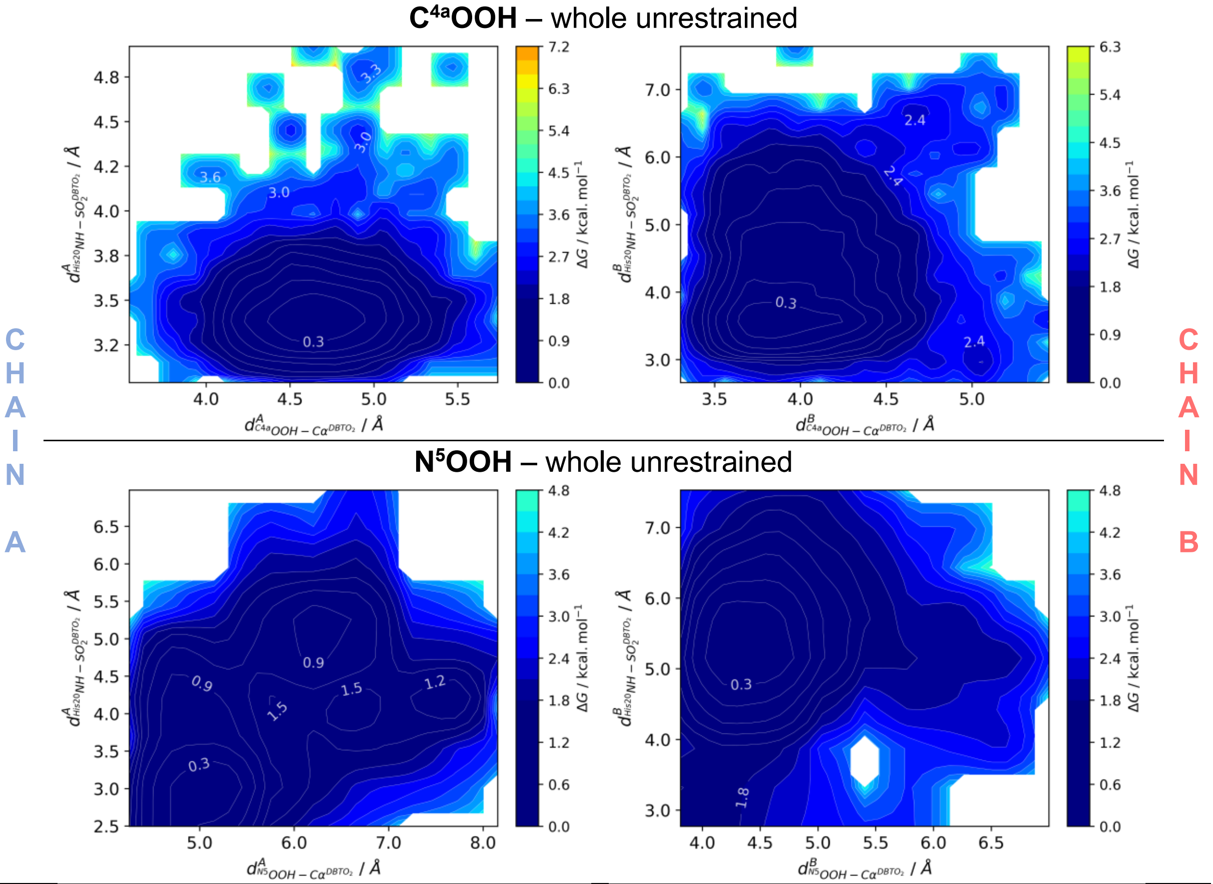

Figure SI 16. 2D-Free energy projection along the distance between the OOH in the FMN cofactor and the Cα1/2 of $DBTO_2$ (proposed as critical under the current mechanistic hypothesis in the literature) and the distance between the NεH of His20 and the $DBTO_2$-sulfone (as mutation of His20 by Phe reduced DszA activity and this is the only conserved polar interaction established by $DBTO_2$ in both models). Each isoenergetic line corresponds to a 0.3 kcal·mol^{-1} difference.

Mechanistic attempts through the C^{4a}OOH model

The QM/MM model consists of a truncated model including DszA residues within 12 Å of the active site of chain A (total of 4299 atoms and –1 charge), where an outer shell of 4 Å was kept frozen. All calculations in the model were ran with Gaussian 16. The QM layer consisted of selected part of residues His20, Asp59, Ser139, Val371, Phe373 and the C4aOOH cofactor, the whole DBTO2 substrate and two water molecules (137 atoms with total zero charge and singlet multiplicity). The summary of the resulting linear transit scans of the mechanistic attempts ran is presented in **Figure SI 17**.

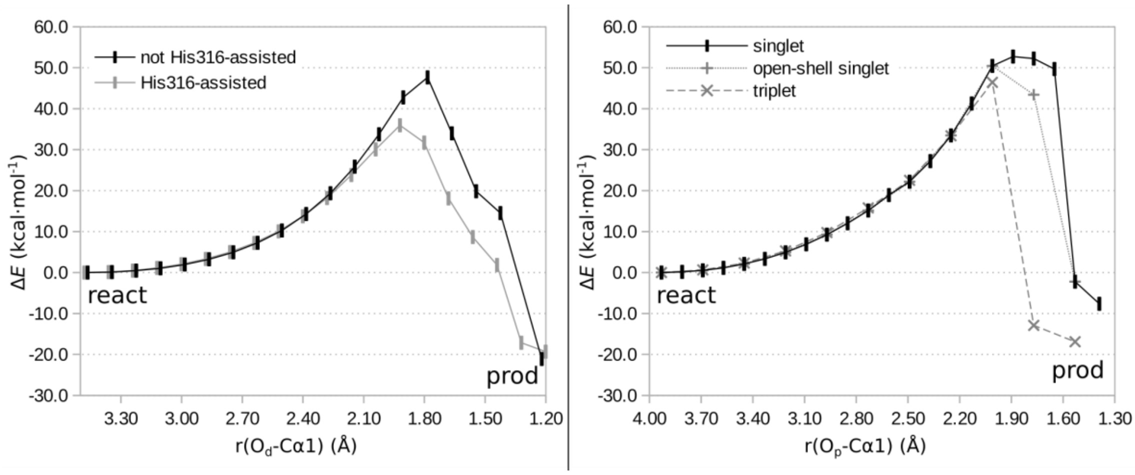

Figure SI 17. Linear transit scans for the hypothesis tested on the truncated QM/MM model for the DszA:C^{4a}OOH:$DBTO_2$ complex: on the left, the formation of the peroxyhemiacetal by decreasing the O_d-Cα1 distance; on the right, the transfer of the hydroperoxyl group by decreasing the O_p-Cα1 distance.

Formation of peroxyhemiacetal intermediate

Once the distance between the distal oxygen of the hydroperoxyl of C^{4a}OOH (O_d) and the closest Cα of $DBTO_2$ shortens, a maximum in which the O_d is linearly aligned with the O_p of C^{4a}OOH and the Cα1 of $DBTO_2$ at distances of 1.96 Å and 1.78 Å, respectively, is observed at about 47.6 kcal·mol^{-1} above the reactant state (**Figure SI 17**, not His316-assisted). Upon formation of the Cα1-O_d bond, O_dH gets deprotonated by the nearby O_p and the Cα1-O_d bond becomes a double bond; there is also a slight cleavage of the Cα1-S at $DBTO_2$ (it elongates to 2.02 Å). The reaction gives rise to an exothermal product (ΔE ~ -20 kcal·mol^{-1}. Although ring opening is observed (**Figure SI 18**, ***prod***), and in addition to the prohibitive energy barrier observed, there seems to be strain preventing rotation of the product molecule around the Cβ1- Cβ2 axis of $DBTO_2$ and only waters are available as closeby proton donors, which would thus likely require a proton relay system from the bulk to complete the 2-hydroxibiphenyl-2-sulfinate (HBPS) product.

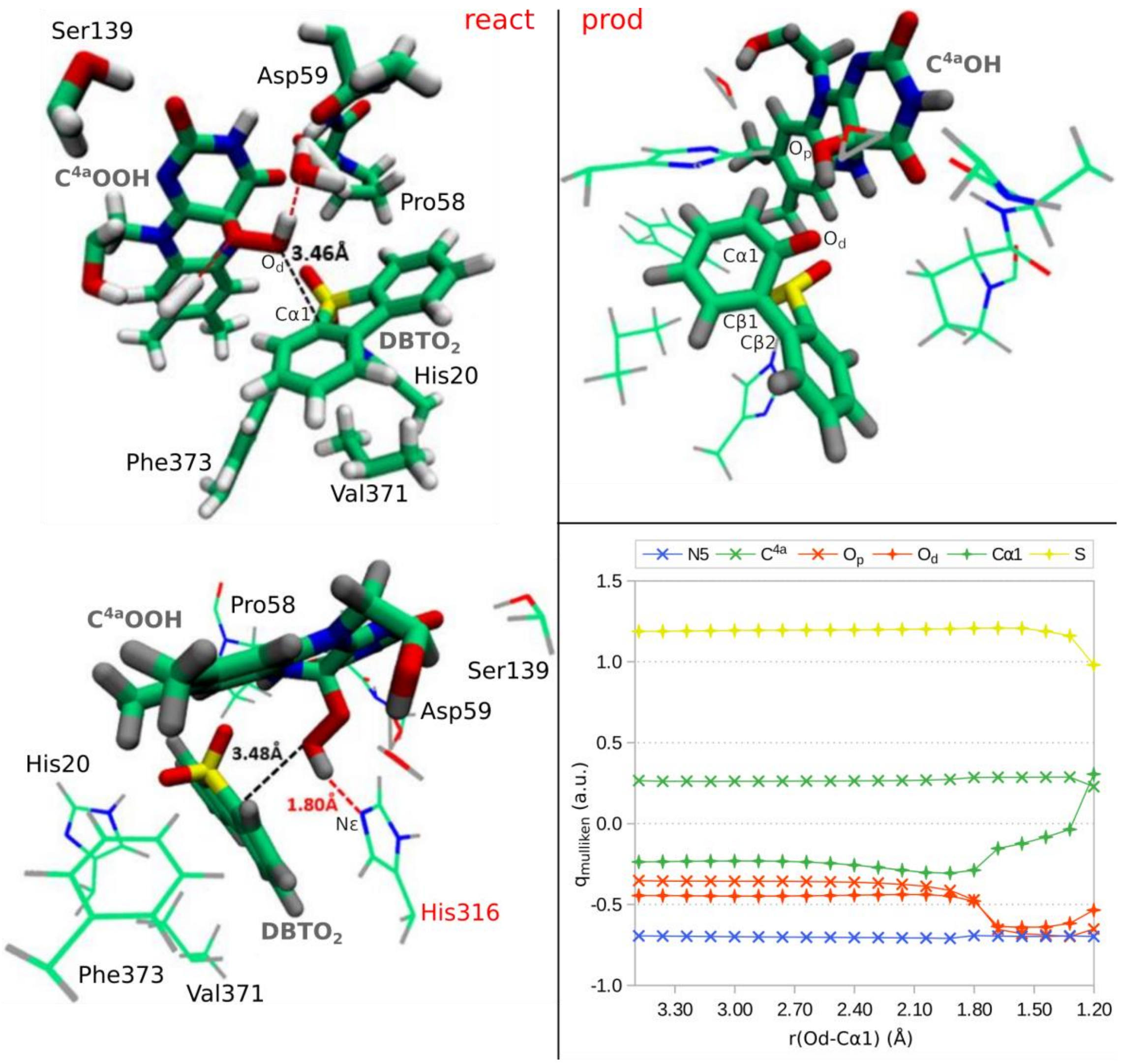

Figure SI 18. The reactant and product states for the mechanistic attempts to form the peroxyhemiacetal intermediate: top and bottom react correspond to the attempt without and with His316 as an assisting base, respectively; bottom right panel depict the Mulliken charges along the His316-assisted attempt for the atoms possibly involved in redox reactions.

Since the 47.6 kcal·mol^{-1} barrier would be prohibitive for the reaction, we also tested the hypothesis for a base-assisted mechanism in which, similarly to the DszC monoxygenase, His316 could play a role as an anchor for the O_dH throughout the O_d transfer to Cα1 of $DBTO_2$ (**Figure SI 17**, His316-assisted). To do so, the sidechain of His316 was included in the QM layer, and linear transit scans were conducted to form a hydrogen bond between the His316-imidazole and the O_dH (**Figure SI 18**, bottom ***react***). A linear transit scan to assess Cα1-O_d bond formation from the new reactant state shows a maximum 36.0 kcal·mol^{-1} above the reactant state, in which the O_d is linearly aligned with the O_p of $C^{4a}OOH$ and the Cα1 of $DBTO_2$ at distances of 1.77 Å and 1.92 Å, and leading to a similar product and reaction energy. Despite that a lower barrier is registered,

this is still prohibitive for efficient catalysis by DszA under biological conditions, and the conclusion of the reaction presents similar problems to the previous attempt.

Formation of the peroxyhemiacetal proposed by Adak and Begley is also not observed in any of the attempts.

Finally, an analysis of the Mulliken charges along the linear transit with the lowest energy barrier (**Figure SI 18**, bottom right panel), shows that the Cα1 of $DBTO_2$ becomes highly electron deficient and most electron density is transferred for the O_p in $C^{4a}OOH$ and the S of $DBTO_2$. In this scenario, more electron donors would be required to reduce the Cα1-O_d double bond instead of the FMN cofactor, and formation of the N5-oxide observed by Adak and Begley is highly unlikely.

Transfer of the hydroperoxyl group to the closest Cα of $DBTO_2$

To assess whether a group transfer of the hydroperoxyl in $C^{4a}OOH$ to the Cα1 of $DBTO_2$ was possible, a linear transit scan along the distance between the O_p of $C^{4a}OOH$ and the Cα1 of $DBTO_2$ was performed (**Figure SI 17**, singlet). The resulting energy profile shows a maximum ~52 kcal·mol^{-1} above the reactant state, in which the Cα1 is adopting a *sp*3 arrangement and the O_p in the OOH group is at 1.89 Å. Once OOH transfer is complete, there is Cα1-S bond cleavage and the ring opening is more pronounced that in the previous studied attempts. In addition, the SO_2 of $DBTO_2$ deprotonates the N5 of $C^{4a}OOH$ suggesting that there is sulfinate formation. The resulting reaction energy is less exothermal than the previously observed (~-8 kcal·mol^{-1}). We also ran single-point energy calculations for open-shell singlet and triplet states, to assess if a spin inversion could contribute for the lowering of the energy barrier for the process (**Figure SI 17**, open-shell singlet and triplet); however, the maximum energy could not go below 45 kcal·mol^{-1}. Instead the single-point energy calculations suggested that the resulting 2'-hydroperoxybiphenyl-2-sulfinate intermediate might be more stable in the triplet spin configuration. Nevertheless, resulting energy results suggest that this pathway is also unlikely.

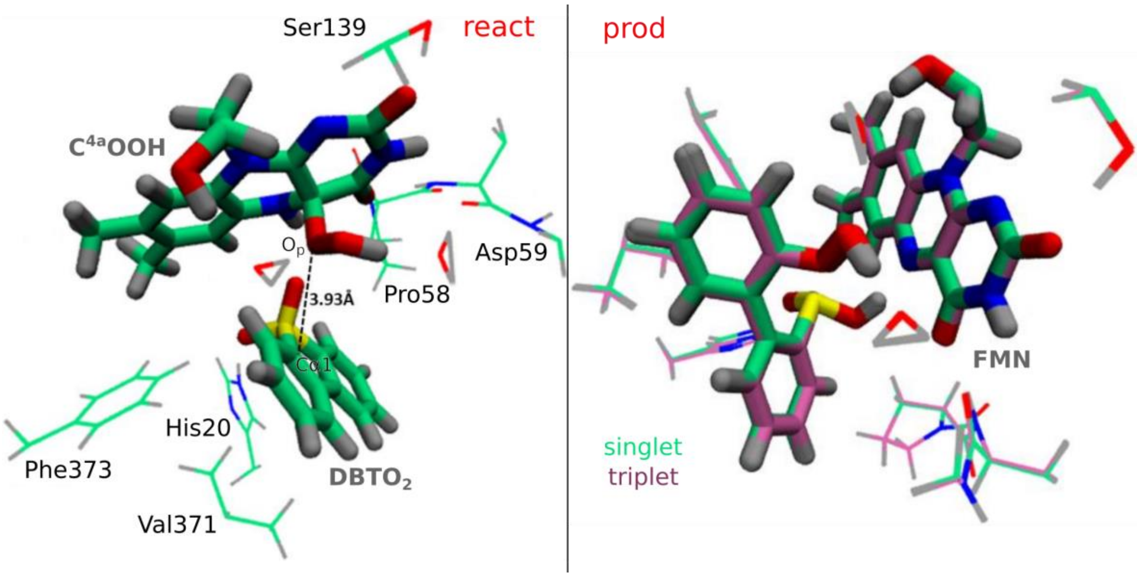

Figure SI 19. The reactant and product states for the mechanistic attempts to transfer the hydroperoxyl group.

Nucleophilic attack of the O_dH group to the closest Cα of $DBTO_2$ with a full QM/MM model

For a direct comparison with the results obtained with the N^5OOH model, we calculated the energy profile of the first step of the C^{4a}OOH pathway using a full QM/MM model (like the one used to study the pathway through N^5OOH, as described on the methodology section on the main text). Defining as reaction coordinate the distance between O_d and the closest Cα of $DBTO_2$ we obtained a simple OH transfer from the cofactor to the substrate concerted with the cleavage of the Cα-S bond. The energy barrier obtained from the freely optimized TS and minima (after an IRC calculation) was 45.4 kcal.mol^{-1}. During the optimization of the INT, the proton bonded to the Od atom spontaneously jumped to the Op atom. These results show this pathway is very unfavorable on DszA.

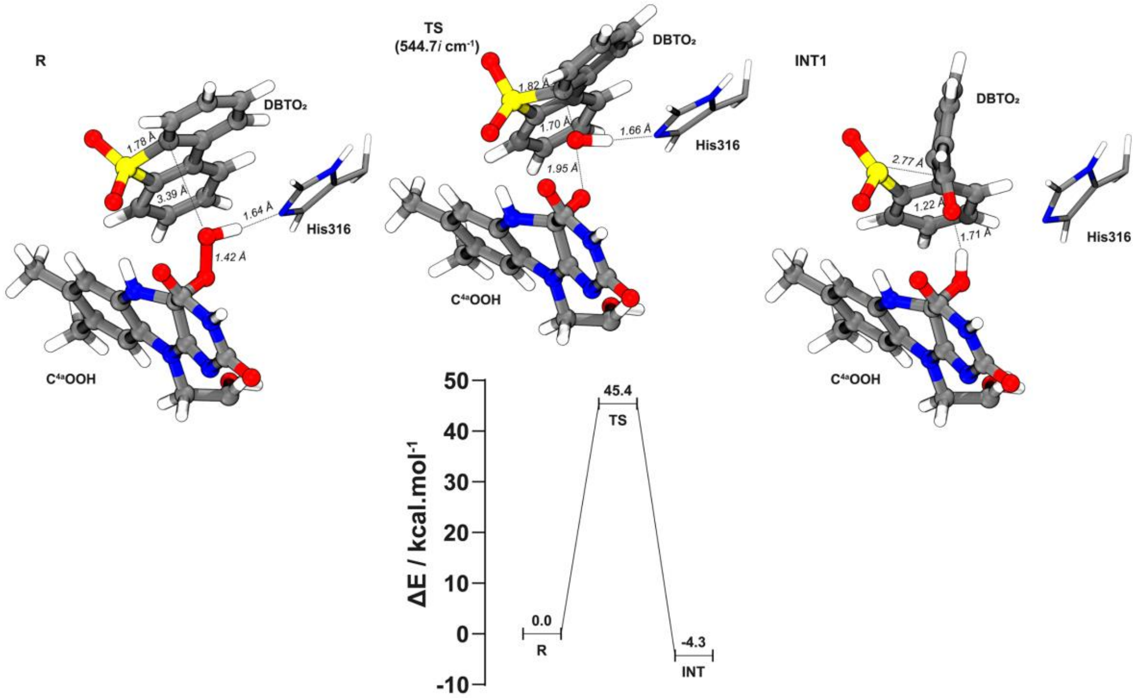

Figure SI. 20. Optimized geometries and energy profile obtained for step 1 of the pathway through $C^{4a}OOH$ with the complete QM/MM model.

Analysis of the electronic configuration of step 2

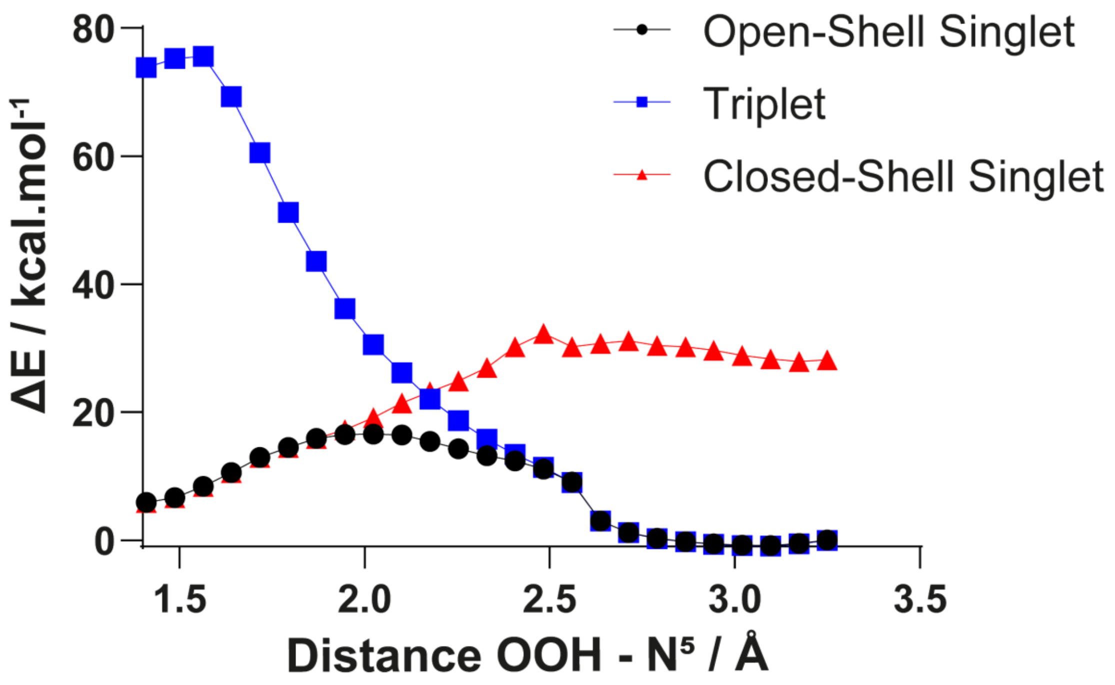

Figure SI. 21. Minimal energy path of the second mechanistic step corresponding to the radical coupling between the protonated superoxide molecule and the semiquinone. The minimal energy path was determined in an open-shell singlet configuration and single point energy calculations were carried out in closed-shell single and triplet spin multiplicities. At the beginning of the step the triplet state is the most

favorable, the energy of the triplet and open-shell singlet remain virtually identical up until a OOH-N^5 distance of around 2.5 Å, from that point onwards the open-shell singlet is the most favorable configuration. After the TS, when the reaction coordinate is 1.99 Å, the open and closed-shell singlet state exhibit the same energy.

Thermochemistry calculations with different density functionals

Table SI 2. Free energies obtained on SP calculations performed with seven different DFT functionals using the optimized geometries obtained at the PBE0-D3BJ/def2-SVP:ff10 level of theory. For all cases we used the def2-TZVPP basis set.

	PBE0	***CAM-B3LYP***	***M06-2X***	***MPWB1K***	***PW6B95***	***PWPB95***	***DSD-PBEB95***
R	0	0	0	0	0	0	0
TS1	1.8	-0.5	3.1	3.5	2.9	2.1	4.0
INT1	-7.0	-10.1	-7.1	-8.2	-6.2	-8.1	-6.0
TS2	11.0	8.8	11.4	11.6	10.6	9.7	14.3
INT2	3.1	-1.0	-2.2	0.2	4.3	-1.3	-2.1
TS3	6.5	3.3	1.9	4.2	7.7	2.5	2.1
INT3	4.6	1.7	-1.1	2.9	4.6	-0.8	-1.3
TS4	9.7	12.3	11.4	14.4	9.4	3.1	2.8
P	-26.7	-30.1	-35.4	-27.8	-27.5	-32.9	-33.6

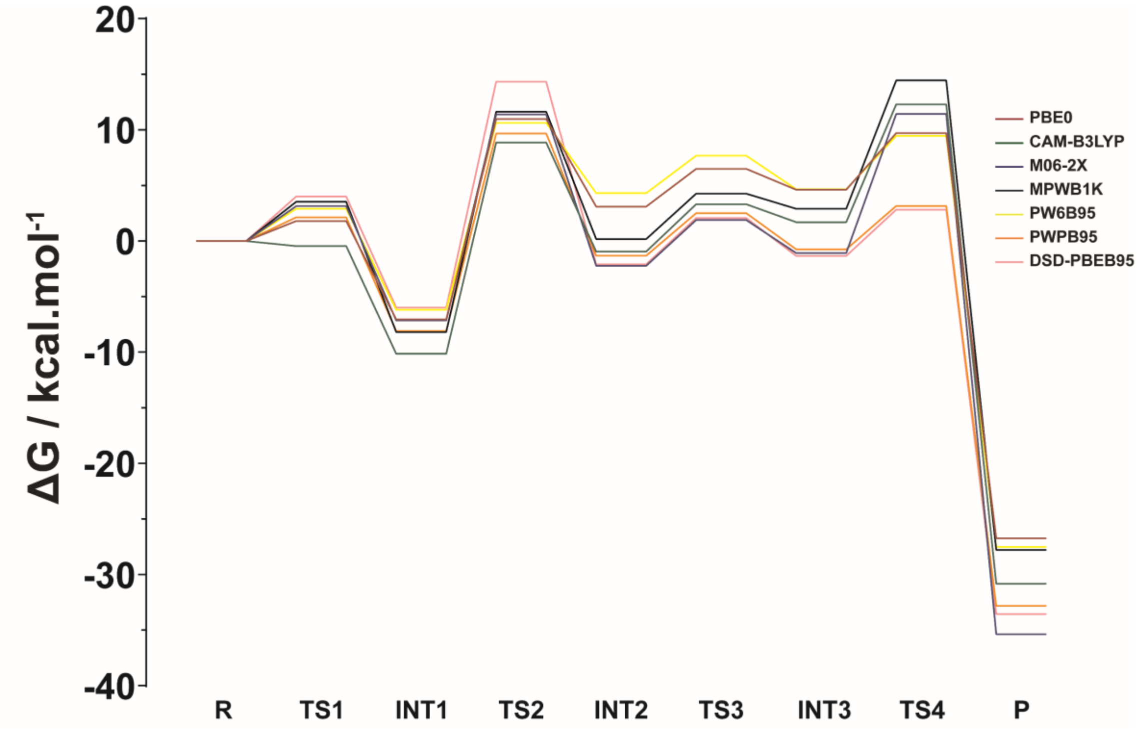

Figure SI 22. Free energy profiles obtained from single-point energy calculations with seven different DFT functionals using the optimized geometries obtained at the PBE0-D3BJ/def2-SVP:ff10 level of theory. For all cases we used the def2-TZVPP basis set.

To analyze the dependence of the obtained energy profile on the choice of the DFT functional, we conducted single-point energy calculations using seven different DFT functionals (PBE0, CAM-B3LYP, M06-2X, MPWB1K, PW6B95, PWPB95 and DSD-PBEB95) on geometries optimized at the PBE0-D3BJ/def2-SVP:ff10 level of theory. All calculations utilized the def2-TZVPP basis set.

The functionals were chosen to cover a wide range of different properties. PBE0, PW6B95 and MPWB1K are hybrid functionals with 25%, 29% and 44% of Hartree-Fock exchange respectively, M06-2X is a hybrid meta-GGA with 54% of Hartree-Fock exchange, CAM-B3LYP is a long-range corrected hybrid functional with 19% and 65% of Hartree-Fock exchange at short and long-range respectively, PWPB95 is a double-hybrid meta-GGA functional with 25% Hartree-Fock exchange, which extends the PW6B95 functional with additional meta-GGA terms and a perturbative correction for the correlation energy based on the second-order Møller-Plesset (MP2) perturbation theory and DSD-PBEB95 which is a double-hybrid DFT functional that employs spin-component scaled MP2 and an empirical dispersion correction parameter.

The energy profiles obtained were similar, although the activation free energies varied from 16.8 $kcal.mol^{-1}$ and 22.6 $kcal.mol^{-1}$. The predicted rate-limiting step also varied between step 2 (predicted by PBE0, PW6B95, PWPB95 and DSD-PBEB95) and step 4 (predicted by CAM-B3LYP and MPWB1K) while M06-2X predicted identical activation energies for both steps (18.5 $kcal.mol^{-1}$).

We chose PWPB95 since the combination of DFT and wavefunction-based methods should result on a more accurate description of electron correlation effects compared to single-hybrid functionals while the long-range dispersion (van der Waals) interactions are corrected with the addition of the D3BJ dispersion correction term. This notion is supported by the excellent performance of PWPB95 in extensive benchmarks [3].

Energy Decomposition Analysis

We conducted activation strain model [4] calculations on the QM region of the catalytic step that corresponds to the hydroxyl transfer from the cofactor to the substrate to further elucidate the reactivity of both $C^{4a}OOH$ and $N^{5}OOH$ intermediates and to elucidate why in DszA, the infrequent pathway through N5OOH is the most favorable one. The system was divided in three fragments, *viz.*, the substrate, the flavin cofactor and the remainder of the protein environment present on the QM layer of the QM/MM claculations. The strain was then evaluated with respect to the geometries of these fragments in the reactants of the step, and the binding energy was further decomposed using the Morokuma-Ziegler Energy Decomposition Analysis. [5] These calculations were performed using the AMS 2023 suite [6], employing the PBE0 functional with the MBD [7] combined with the TZ2P basis set.
The strain contribution dominates overwhelmingly the differences observed (**Table SI 3**). For the C4a pathway, the strain contributes 50 $kcal.mol^{-1}$ to the activation energy barrier while only 16.61 $kcal.mol^{-1}$ for the N5 pathway. As expected, the strain contribution is bigger on the flavin cofactor fragment (35.24 for the C4a pathway vs. 7.18 for the N5 pathway). This difference in strain of the flavin fragment can be explained by the fact that on the N5 pathway, N5 can donate its lone pair to facilitate the cleavage of the Od-Op bond while this is much more difficult on the C4 pathway.

The differences in the binding energies between the fragments on the two different pathways is much smaller; the contribution to the calculated activation energy is -6.56 and -5.09 for the C4 and N5 pathway respectively. The individual components to this binding energy are very different for the two pathways, but that is a natural consequence of the very different charge distributions for the two pathways: in the N5 pathway, the flavin fragment is negatively charged, while the environment is positively charged, whereas in the C4a pathway all fragments are neutral.

Table SI 3. ASM and EDA analysis for the rate limiting step of the reaction on both possible pathways. All energy components are in kcal.mol^{-1}.

	**Strain Cofactor**	**Strain Substrate**	**Strain Enzyme**	**ΔE_{Pauli}**	**ΔE_{Elstat}**	**ΔE_{Orb}**	**ΔE_{Disp}**	**ΔE_{Efield}**	**ΔE_{Bind}**	**$\Delta E_{Strain+Bind}$**
N^5 pathway										
INT3	0.00	0.00	0.00	0.00	0.00	0.00	0.00	0.00	0.00	0.00
TS4	7.18	8.36	1.07	93.54	-57.46	-47.57	-0.50	6.90	-5.09	11.52
Product	25.67	115.67	5.24	464.49	-241.98	-396.12	-0.06	4.35	-169.32	-39.35
C^{4a} pathway										
R	0.00	0.00	0.00	0.00	0.00	0.00	0.00	0.00	0.00	0.00
TS1	35.24	15.58	-0.80	160.26	-69.46	-99.21	-2.46	4.31	-6.56	43.46
INT1	84.77	123.76	-0.22	783.66	-311.70	-682.79	1.78	-3.12	-212.17	-53.88

References

(1) Wiederstein, M.; Sippl, M. J. ProSA-web: interactive web service for the recognition of errors in three-dimensional structures of proteins. *Nucleic Acids Res* **2007**, *35* (Web Server issue), W407-410. DOI: 10.1093/nar/gkm290 From NLM.

(2) Sousa, J. P. M.; Ferreira, P.; Neves, R. P. P.; Ramos, M. J.; Fernandes, P. A. The bacterial 4S pathway – an economical alternative for crude oil desulphurization that reduces CO2 emissions. *Green Chem.* **2020**, *22* (22), 7604-7621. DOI: 10.1039/D0GC02055A.

(3) Goerigk, L.; Grimme, S. A thorough benchmark of density functional methods for general main group thermochemistry, kinetics, and noncovalent interactions. *Phys. Chem. Chem. Phys.* **2011**, *13* (14), 6670-6688, 10.1039/C0CP02984J. DOI: 10.1039/C0CP02984J. Goerigk, L.; Grimme, S. Efficient and Accurate Double-Hybrid-Meta-GGA Density Functionals—Evaluation with the Extended GMTKN30 Database for General Main Group Thermochemistry, Kinetics, and Noncovalent Interactions. *J. Chem. Theory Comput.* **2011**, *7* (2), 291-309. DOI: 10.1021/ct100466k.

(4) Vermeeren, P.; Hamlin, T. A.; Bickelhaupt, F. M. Chemical reactivity from an activation strain perspective. *Chem. Commun.* **2021**, *57* (48), 5880-5896. DOI: 10.1039/D1CC02042K.

(5) Bickelhaupt, F. M.; Baerends, E. J. Kohn-Sham Density Functional Theory: Predicting and Understanding Chemistry. In *Rev. Comput. Chem.*, Lipkowitz, K. B., Boyd, D. B. Eds.; John Wiley & Sons, Ltd, 2000; pp 1-86. Ziegler, T.; Rauk, A. A theoretical study of the ethylene-metal bond in complexes between copper(1+), silver(1+), gold(1+), platinum(0) or platinum(2+) and ethylene, based on the Hartree-Fock-Slater transition-state method. *Inorganic Chemistry* **1979**, *18* (6), 1558-1565. DOI: 10.1021/ic50196a034. Ziegler, T.; Rauk, A. Carbon monoxide, carbon monosulfide, molecular nitrogen, phosphorus trifluoride, and methyl isocyanide as .sigma. donors and .pi. acceptors. A theoretical study by the Hartree-Fock-Slater transition-state method. *Inorganic Chemistry* **1979**, *18* (7), 1755-1759. DOI: 10.1021/ic50197a006. Kitaura, K.; Morokuma, K. A new energy decomposition scheme for molecular interactions within the Hartree-Fock approximation. *International Journal of Quantum Chemistry* **1976**, *10* (2), 325-340. DOI: https://doi.org/10.1002/qua.560100211. Mitoraj, M. P.; Michalak, A.; Ziegler, T. On the Nature of the Agostic Bond between Metal Centers and β-Hydrogen Atoms in Alkyl Complexes. An Analysis Based on the Extended Transition State Method and the Natural Orbitals for Chemical Valence Scheme (ETS-NOCV). *Organometallics* **2009**, *28* (13), 3727-3733. DOI: 10.1021/om900203m. Mitoraj, M. P.; Michalak, A.; Ziegler, T. A Combined Charge and Energy Decomposition Scheme for Bond Analysis. *J. Chem. Theory Comput.* **2009**, *5* (4), 962-975. DOI: 10.1021/ct800503d.

(6) te Velde, G.; Bickelhaupt, F. M.; Baerends, E. J.; Fonseca Guerra, C.; van Gisbergen, S. J. A.; Snijders, J. G.; Ziegler, T. Chemistry with ADF. *J. Comput. Chem.* **2001**, *22* (9), 931-967. DOI:

https://doi.org/10.1002/jcc.1056. *ADF 2023.1, SCM, Theoretical Chemistry, Vrije Universiteit, Amsterdam, The Netherlands, http://www.scm.com*; 2023. (accessed. Fonseca Guerra, C.; Snijders, J. G.; te Velde, G.; Baerends, E. J. Towards an order-N DFT method. *Theor. Chem. Acc.* **1998**, *99* (6), 391-403. DOI: 10.1007/s002140050353.
(7) Ambrosetti, A.; Reilly, A. M.; DiStasio, R. A., Jr.; Tkatchenko, A. Long-range correlation energy calculated from coupled atomic response functions. *J Chem Phys* **2014**, *140* (18), 18a508. DOI: 10.1063/1.4865104 From NLM.
